# Human Hematopoietic Stem Cells Enhance Maturational Differentiation of hiPSC-Derived Cardiomyocytes on Xeno-Free MatriClone-Plastic via EGFR/MAPK/ERK Signaling Pathway

**DOI:** 10.3390/ph19060964

**Published:** 2026-06-22

**Authors:** Ke Sun, Hongmei Li, Lu Wang, Ting Wang, Guangrui Huang, Anlong Xu

**Affiliations:** 1 School of Life Science, Beijing University of Chinese Medicine, Beijing 100020, China; 20210941105@bucm.edu.cn (K.S.); hongmei.li@bucm.edu.cn (H.L.); 20240931154@bucm.edu.cn (L.W.); 2 The First Clinical College, Inner Mongolia Medical University, Hohhot 010000, China; wt@bucm.edu.cn; 3 Hong Kong Institute of Advanced Studies, Sun Yat-sen University, Hong Kong 999077, China

**Keywords:** cardiac differentiation, epidermal growth factor receptor/mitogen-activated protein kinase/extracellular signal-regulated kinase signaling pathway, human hematopoietic stem cells, human induced pluripotent stem cells, xeno-free

## Abstract

**Background/Objectives**: Only substantial quantities of xeno-free human induced pluripotent stem cell (hiPSC)-derived cardiomyocytes (CMs) (hiPSC-CMs) with stable quality and structural and functional maturity can meet the demand for cardiac cell therapy. The use of xeno-free microcarriers can significantly increase cell yield. Co-culturing with hematopoietic stem cells (HSCs) simulates the environment in vivo and has a necessary impact on the development of CMs. However, no microcarrier-based protocol for xeno-free hiPSC-CM culture has yet been established, and the effects of HSCs on CM development and their underlying mechanisms remain unclear. Therefore, this study aims to investigate these issues. **Methods**: We used a xeno-free microcarrier (plastic) culture system coated by a defined xeno-free matrix (MatriClone) to expand hiPSCs and hiPSC-CMs with human hematopoietic stem cells (hHSCs). Using RNA sequencing (RNA-seq), cytokine assay, and various cellular molecular techniques, we investigated the role of hHSCs in cardiac differentiation and maturation, and underlying mechanisms. **Results**: hiPSCs were evenly distributed on the surface of plastic coated with 1 μg/cm^2^ MatriClone (MatriClone-Plastic), increasing and sustaining pluripotency marker levels. Directed differentiation of hiPSCs on 1 μg/cm^2^ MatriClone-Plastic induced a larger number of CMs, and the level of cardiac differentiation was also significantly improved. When hHSCs were co-cultured with cells at the cardiac progenitor cell stage, results from electron microscopy, electrophysiology, and qPCR showed that hiPSC-CMs significantly promoted cardiac structural and functional maturation. The co-cultured hHSCs released multiple cytokines that were changed dynamically at different time points, and that were highly likely to activate the epidermal growth factor receptor (EGFR)/mitogen-activated protein kinase (MAPK)/extracellular signal-regulated kinase (ERK) signaling pathway to promote cardiac development and maturation. **Conclusions**: hHSCs can efficiently promote differentiation and maturation of xeno-free hiPSC-CMs on MatriClone-Plastic via the EGFR/MAPK/ERK signaling pathway.

## 1. Introduction

Human cardiomyocytes (CMs) have limited regenerative capacity; however, human pluripotent stem cells (hPSCs), including human embryonic stem cells (hESCs) and human induced pluripotent stem cells (hiPSCs), are considered an unlimited source for generating human cells, including CMs, due to their self-renewal and directed differentiation capabilities [1]. This potential has also facilitated the development of novel cell therapies for organ regeneration, leading to a significant increase in related clinical research [2]. In addition, relevant human investigations have shown the effectiveness and safety of hiPSC-derived CMs (hiPSC-CMs) for the treatment of heart failure [3]. Therefore, transplantation of hiPSC-CMs may be a promising strategy to improve the function of failing myocardium. To date, several protocols and techniques have been developed to differentiate hPSCs into CMs, with the aim of yielding functionally and structurally mature CMs [4]. The majority of hiPSC-CMs are produced through adherent static cell culture systems, which are low-cost and easy to handle. Problems such as poor cell yields, insufficient quality assessment of hiPSC-CMs, and inter-batch effects have limited experiment repeatability and clinical viability.

Employing suspension culture and scalable reactor systems can provide sufficient, batch-consistent quantities of differentiation-competent or differentiated cells to meet large-scale demand. hiPSC-CMs acquired from suspension culture differentiation have been successfully used to model heart disease or to test novel molecular therapeutics [5,6]. In particular, attaching cells to the microcarrier surface provides a larger surface area and homogeneous culture environment for cell adhesion and growth. It is beneficial to retain cell phenotypes and decrease contact inhibition in classic monolayer culture, which promotes cell differentiation and proliferation [7]. However, most protocols are based on the use of animal-derived extracellular matrix (ECM) extracts of unspecified composition (such as Matrigel or Geltrex) for use as a surface for cell attachment to microcarriers. Meanwhile, the differentiation of xeno-free hiPSC-CMs has yet to be explored in a systematic differentiation method.

In addition to this, cells exist in a 3D environment in vivo. Monolayer cell culture and its culture conditions are easy to control, but there is a substantial difference between the environment in vivo, resulting in low structural and functional maturation of most hiPSC-CMs, making it difficult to meet clinical needs. Differentiation of organoids is time-consuming and expensive. A consistent and uniform differentiation process, particularly for cardiac organoids, has yet to be developed. It is challenging to maintain the form, size, and developmental status of a single cell in vitro for an extended period of time. Cell co-culture technology can effectively compensate for the limitations of monolayer culture and organoids, and simulate the environment in vivo, thereby facilitating a more comprehensive investigation into cell–cell interactions and the induction of cell differentiation [8]. In recent years, with the development of single-cell RNA-seq and single-nucleus RNA-seq, it has become increasingly evident that the complex intercellular communication and interactions between CMs and non-CMs have a critical impact on normal heart development. It was identified by single-cell RNA-seq that multiple cell types, such as cardiomyocyte subpopulations and blood cells, in embryonic hearts between 8 and 17 post-conception weeks, play a potential role in cardiac development. Communication and interaction between CMs and blood cells were also found to increase significantly over time, but when these cellular communications play an active role and what their specific mechanisms are still need to be further clarified [9,10,11]. As the most primitive hematopoietic cells, hematopoietic stem cells (HSCs) possess the ability to differentiate into all types of blood cells due to their multipotent differentiation potential [12,13,14,15]. HSCs undergo directed differentiation into a complete family of cells, known as the hematopoietic lineage, and as a result, multiple transcription factors that regulate hematopoietic lineage differentiation are highly expressed in HSCs [13,16]. Earlier studies both in vivo and in stem cell models have demonstrated that various progenitor cells interact with each other and develop collaboratively during the early stage of embryonic development, and that both hematopoietic and cardiac lineages derive from the mesoderm, with their development being closely related and mutually regulated [16,17,18]. Studies have shown that the differentiated products of HSCs can promote the proliferation, maturation, and functional integration of CMs [19,20,21,22,23,24]. To date, no direct involvement of HSCs in cardiac development has been revealed, nor have co-cultures of HSCs with hiPSC-CMs been investigated.

To address the aforementioned issue, we chose a suitable xeno-free matrix (Matriclone) and microcarrier (Plastic), optimized the cardiac differentiation protocol established by our own laboratory [25], and implemented a suspension culture system based on the use of MatriClone-coated Plastic for hiPSC scalable expansion and cardiac differentiation to maximize cell yield in the same area. We also co-cultured human HSCs (hHSCs) with hiPSC-CMs at the progenitor cell stage of cardiac differentiation and harvested large amounts of high-purity CMs, indicating that suspension co-cultured induced CMs have better physiological properties than static independently induced CMs. To further investigate the mechanism, we analyzed specific signaling pathways in co-cultured induced hiPSC-CMs using RNA sequencing (RNA-seq). The cytokine assay identified cytokines that were highly expressed in the cell supernatant, and we verified the above conclusions using the Protein Phosphorylation Assay and Quantitative Real-time PCR (qPCR). This study revealed the epidermal growth factor receptor (EGFR)/mitogen-activated protein kinase (MAPK)/extracellular signal-regulated kinase (ERK) signaling pathway as key for hHSCs to promote cardiac differentiation and maturation.

Previous studies have only optimized one of these three elements, such as developing the xeno-free matrix or exploring microcarrier culture. Single-element optimization can only partially meet the requirements of clinical translation. Only by combining the xeno-free matrix, the microcarrier culture, and co-culture with hHSCs can our platform simultaneously meet the three core requirements for clinical translation: safety (xeno-free), scalability (large-scale culture using microcarriers), and efficacy (functionally mature CMs). In summary, this study combines these three complementary elements, the xeno-free matrix, the xeno-free microcarrier, and hHSCs for co-culture, to achieve the dual goals of large-scale xeno-free production and functional maturation of hiPSC-CMs, which cannot be achieved by any single element alone.

## 2. Results

### 2.1. MatriClone, a Xeno-Free Matrix, Promoted Long-Term hiPSC Maintenance and Cardiac Differentiation In Vitro

**Figure 1:**
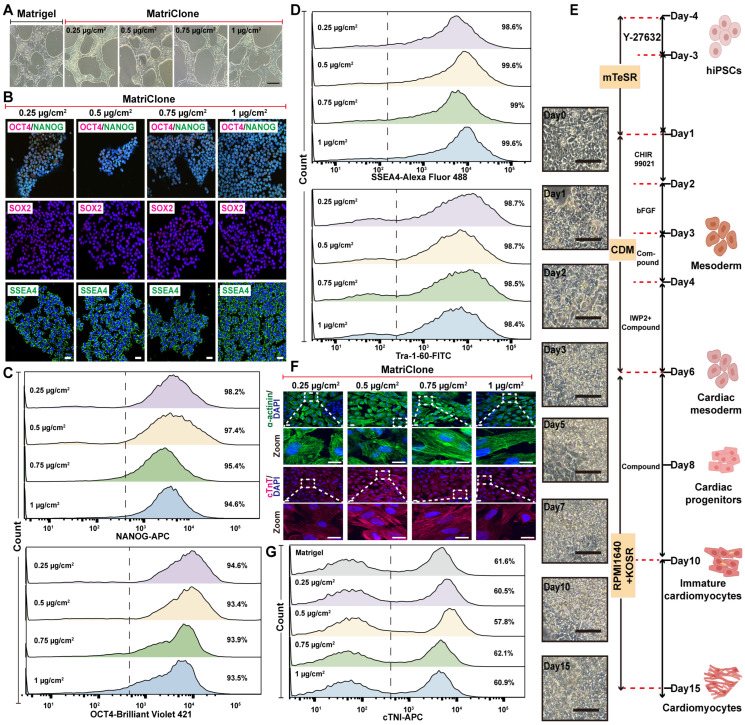
The potential for hiPSC expansion and cardiac differentiation on MatriClone. (**A**) Representative photos of colonies cultured on Matrigel and on MatriClone at densities between 0.25 and 1 μg/cm^2^; scale bars = 100 μm. (**B**) Representative immunofluorescence images of NANOG, OCT4, SOX2, and SSEA4 expression in hiPSCs on MatriClone at densities ranging from 0.25 to 1 μg/cm^2^ for ten consecutive passages. Cells were counterstained with DAPI to visualize cell nuclei. Scale bars = 25 μm. (**C**,**D**) Flow cytometry was used to detect the pluripotency of hiPSCs over ten passages on MatriClone at densities ranging from 0.25 to 1 μg/cm^2^. Representative histograms of hiPSCs showed the proportion of NANOG, OCT4, SSEA4, and TRA-1-60-expressing cells. (**E**) Schematic representation of the differentiation procedure and sequential morphological changes (Day 4–15) of hiPSC differentiation into CMs induced by the saponin^+^ compound. Scale bars = 100 μm. (**F**) Representative immunofluorescence images of CM markers α-actinin and cTNT in Day 15 hiPSC-CMs on MatriClone at densities ranging from 0.25 to 1 μg/cm^2^. Scale bars = 25 μm. (**G**) Flow cytometry was used to detect the positive rate of unpurified hiPSC-CMs. Representative histogram of hiPSC-CMs showed the proportion of cTnI-expressing cells in Day 15 hiPSC-CMs on MatriClone at varying densities, and on Matrigel.

To date, only a few xeno-free matrices have been produced to support hiPSC culture. Based on several factors, including cost, we chose MatriClone, which consists mainly of recombinant laminin-511 E8 fragments, for hiPSC line U1 (urothelial cell-derived) culture and subsequent differentiation of hiPSCs into CMs. We developed numerous densities based on the manufacturer’s instructions to determine the best coating density for routine usage. There were no significant variations in cell shape between hiPSCs on MatriClone at varying densities and Matrigel. Furthermore, they were all able to form compact colonies with distinct borders and high nucleus-to-cytoplasm ratios (Figure 1A). hiPSCs were next grown on MatriClone for ten consecutive passages, and the expression of the pluripotency markers was assessed by immunofluorescence (Figure 1B). The fluorescence images indicated that different densities of MatriClone could maintain the undifferentiated state of hiPSCs. Flow cytometry results also showed that over 95% of cells highly expressed NANOG, octamer-binding transcription factor 4 (OCT4), stage-specific embryonic antigen-4 (SSEA4), and TRA-1-60 (Figure 1C,D). Meanwhile, the same batch of hiPSCs was differentiated toward all three germ layers, and the immunofluorescence results showed that the differentiated cells all expressed markers of different germ layers (Supplementary Figure S1), showing excellent pluripotency.

Next, hiPSCs on MatriClone at densities ranging from 0.25 to 1 μg/cm^2^ were differentiated into CMs using an optimized protocol independently developed by our research team (Figure 1E) [25]. On the 7th day, some of the cells appeared to be beating, and on the 15th day, most of them transformed into short columnar mononuclear cells and showed regular beating, indicating that the CMs had been developed (Supplementary Video S1–S5). We did not apply hiPSC-CM enrichment methods [26] at the end of the cardiac differentiation protocol. Immunofluorescence staining revealed that CMs at densities ranging from 0.25 to 1 μg/cm^2^ all expressed the CM markers α-actinin and cardiac Troponin T (cTNT) (Figure 1F), but did not express pluripotency markers (Supplementary Figure S2A). Flow cytometry studies further demonstrated that, as compared to Matrigel, there were no significant variations in the proportions of cardiac Troponin I (cTNI)-expressing cells on MatriClone at densities ranging from 0.25 to 1 µg/cm^2^ (Figure 1G).

These results all suggested that MatriClone at densities ranging from 0.25 to 1 μg/cm^2^ was able to maintain good pluripotency of hiPSCs, allowing for long-term culture and the differentiation of CMs in vitro.

### 2.2. Plastic Coated with 1 μg/cm^2^ MatriClone Allowed for Long-Term hiPSC Maintenance and Cardiac Differentiation In Vitro

**Figure 2:**
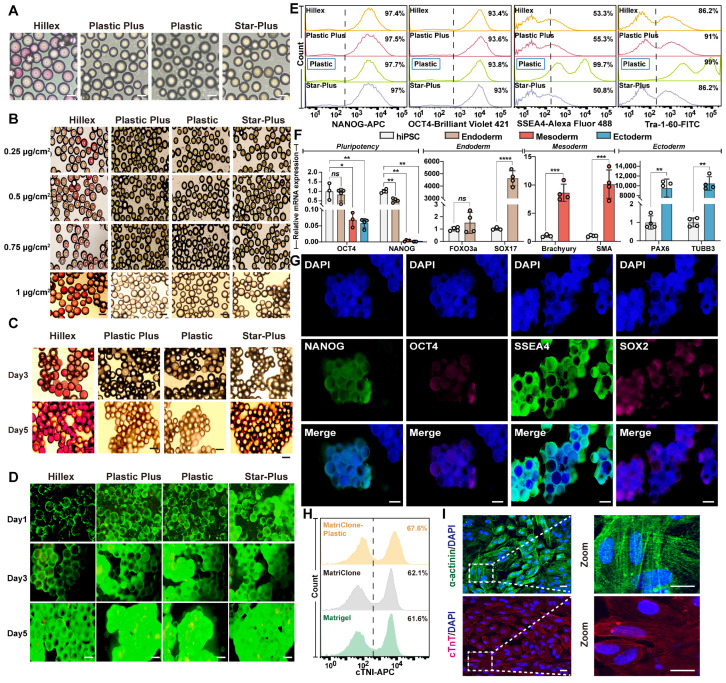
hiPSC expansion and cardiac differentiation on MatriClone-coated microcarriers. (**A**) Representative images of hiPSCs on four xeno-free microcarriers not coated with MatriClone under light microscope (expansion Day 1), scale bars = 200 μm. (**B**) Representative images of hiPSCs on four xeno-free microcarriers coated with MatriClone at densities ranging from 0.25 to 1 μg/cm^2^ under light microscope (expansion Day 1), scale bars = 200 μm. (**C**) Representative images of hiPSCs on four microcarriers coated with 1 μg/cm^2^ MatriClone in a static condition after 3, and 5 days of culture, scale bars = 200 μm. (**D**) After 1, 3, and 5 days of culture, Calcein AM/PI Double Staining was used to detect hiPSC viability on four microcarriers coated with 1 μg/cm^2^ MatriClone (Green: live; Magenta: dead), scale bars = 200 μm. (**E**) Representative histograms of hiPSCs showing the proportion of NANOG, OCT4, SSEA4, and Tra-1-60-expressing cells for ten consecutive passages on four microcarriers coated with 1 μg/cm^2^ MatriClone. (**F**) qPCR analysis was used to characterize the differentiation potential of three germ layers of hiPSCs on 1 μg/cm^2^ MatriClone-Plastic. Values are determined relative to actin and presented as fold change relative to the expression in hiPSCs, which is set as 1. Points represent biological replicates for each independent differentiation (*n* = 3–4). (**G**) Representative immunofluorescence images of pluripotency marker expression in hiPSCs on 1 μg/cm^2^ MatriClone-Plastic, scale bars = 200 μm. (**H**) Representative histograms of hiPSC-CMs show the proportion of cTnI-expressing cells in Day 15 hiPSC-CMs on Matrigel, 1 μg/cm^2^ MatriClone, and 1 μg/cm^2^ MatriClone-Plastic. (**I**) Representative immunofluorescence images of CM markers α-actinin and cTNT on 1 μg/cm^2^ MatriClone-Plastic, scale bars = 25 μm. All data are expressed as mean ± SD. Normality testing was performed using Shapiro–Wilk tests before selecting statistical tests as appropriate. OCT4 in (**F**) was analyzed by nonparametric testing; NANOG in (**F**) was analyzed by one-way ANOVA; other data in (**F**) were analyzed by two-sided Student’s *t*-tests. * *p* < 0.05, ** *p* < 0.01, *** *p* < 0.001, **** *p* < 0.0001, ns, no significance.

To cultivate hiPSCs in a self-renewing and undifferentiated state, an appropriate ECM is required. We have proven that microcarriers alone are insufficient to elicit hiPSC attachment (Figure 2A), so it is necessary to coat microcarriers with matrix [27]. After proving MatriClone’s ability to sustain long-term hiPSC culture, this matrix was employed to coat four distinct commercially available non-animal microcarriers (SoloHill) (Supplementary Table S1), and subsequently implemented a scalable culture.

As described in Section 4, the hiPSCs were seeded onto the four microcarriers coated with MatriClone (MatriClone-microcarriers) at densities between 0.25 and 1 μg/cm^2^ in low adherence 24-well plates at a seeding density of 5 × 10^4^ cells/cm^2^. They were then gently shaken to reduce cell loss due to adherence to the plate surface and enhanced cell-microcarrier interactions. After 1 day of seeding, only attached cells were clearly visible on the 1 μg/cm^2^ MatriClone-microcarriers (Figure 2B). The hiPSC-microcarrier culture reached confluence 3–5 days after seeding, with over 90% of the microcarriers coated with hiPSCs (Figure 2C,D). At this point, the outer layer of cells began to dissociate, and many dead cells were observed floating in the culture. Microcarriers have a high surface-to-volume ratio, allowing for highly concentrated cell cultures, which is required in up-scaled culture systems. Compared to hiPSC culture on conventional MatriClone-coated wells, the cell yielded from the four microcarrier cultures was nearly twice as high (Supplementary Figure S3A). In this study, hiPSCs were continuously cultured on microcarriers for up to 10 passages. Flow cytometry was used to confirm the undifferentiated condition of hiPSCs after 10 passages on 1 μg/cm^2^ MatriClone-microcarriers. Pluripotency markers such as OCT4, NANOG, SSEA4, and TRA-1-60 were detected. The results showed that only plastic was effective in maintaining hiPSC pluripotency (Figure 2E). In addition, differentiation of three germ layers (Figure 2F) and immunofluorescence staining (Figure 2G) were performed on the same batch of hiPSCs cultured on 1 μg/cm^2^ MatriClone-Plastic.

Then, the feasibility of employing 1 μg/cm^2^ MatriClone-Plastic to differentiate hiPSCs into CMs was initially investigated. hiPSCs were seeded onto 1 μg/cm^2^ MatriClone-Plastic at a seeding density of 2.23 × 10^5^ cells/cm^2^ and cultured for 4 days. It was found that the cell density reached a plateau after 2 days of culturing, and the cell concentration or cells on the beads did not increase any further due to surface area restriction (Supplementary Figure S3B). Therefore, hiPSCs were expanded on 1 μg/cm^2^ MatriClone-Plastic for 2 days to generate a confluent monolayer of cells, followed by cardiac differentiation initiation (Supplementary Figure S3C). A cellular beating condition appeared on the 8th day and persisted until the 15th Day (Supplementary Video S6). We did not apply hiPSC-CM enrichment methods [26] at the end of the cardiac differentiation protocol. Flow cytometry results also showed that the proportions of cTNI-expressing cells on 1 μg/cm^2^ MatriClone-Plastic were 67.6%, which was substantially higher than that on Matrigel and MatriClone (Figure 2H and Figure S3D). Immunofluorescence staining revealed that the hiPSC-CMs on the 15th day exhibited the CM markers α-actinin and cTNT on 1 μg/cm^2^ MatriClone-Plastic (Figure 2I). Similarly, as compared to differentiation on MatriClone and Matrigel, cell yield from MatriClone-Plastic cultures was, on average, twice as high (Supplementary Figure S3E). Meanwhile, we used another hiPSC line B1 derived from blood cells on 1 μg/cm^2^ MatriClone-Plastic for cell expansion and cardiac differentiation, with similar results in fluorescence staining and flow cytometry (Supplementary Figure S4).

Taken together, 1 μg/cm^2^ MatriClone-Plastic culture yielded a higher fraction of hiPSCs and hiPSC-CMs, which was suitable for further research.

### 2.3. Co-Culture of Cardiac Differentiation with hHSCs on MatriClone Increased the Proportion of hiPSC-CMs and Drove Their Maturation

Meanwhile, to achieve the co-culture of cardiac differentiation and hHSCs to yield more mature CMs, we explored the best conditions for co-culture on 1 μg/cm^2^ MatriClone. Before co-culture, we assessed the cell viability and surface markers of hHSCs (human bone-marrow-derived). Flow cytometry results showed that 96% of the cells had high cell viability (Supplementary Figure S5A), with 78.1% of hHSCs expressing both CD34 and CD90 (Supplementary Figure S5B). Furthermore, the proportion of hHSCs with double-negative expression of CD38 and CD45RA was as high as 99.9% (Supplementary Figure S5C), indicating that hHSCs were multipotent progenitor cells. Subsequently, we co-cultured hiPSC lines U1 with these hHSC lines.

**Figure 3:**
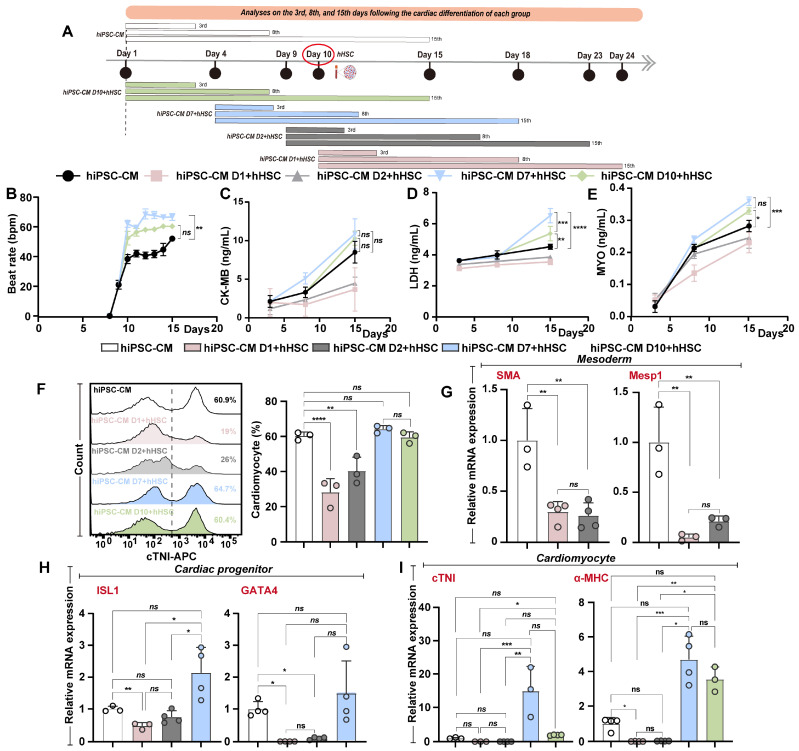
Exploration of the optimal time for co-culture of hHSCs and cardiac differentiation. (**A**) Schematic representation of the experiment for the best time of co-culture of hHSCs and cardiac differentiation. (**B**) The beat rate of cells in each group was recorded every day from Day 8 (*n* = 3). (**C**–**E**) The levels of CK-MB, LDH, and MYO of cells in each group were detected by ELISA on the 3rd, 8th, and 15th day (*n* = 3–4). (**F**) Representative histogram of hiPSC-CMs showed the proportion of cTnI-expressing cells in Day 15 hiPSC-CMs. Flow cytometry was used to determine the proportion of cTnI-expressing cells in Day 15 hiPSC-CMs (*n* = 3). (**G**) qPCR analysis was used to characterize mesoderm markers SMA and Mesp1 in each group on the 3rd day (*n* = 3–4). (**H**) qPCR analysis was used to characterize cardiac progenitor markers ISL1 and GATA4 in each group on the 8th day (*n* = 3–4). (**I**) qPCR analysis was used to characterize CM markers cTNI and α-MHC in each group on the 15th day (*n* = 3–4). All data were expressed as mean ± SD. The hiPSC-CM group was used as a negative control. Normality testing was performed using Shapiro–Wilk tests before selecting statistical tests as appropriate. Data in (**B**,**I**) were analyzed by nonparametric testing; ISL1 in (**H**) was analyzed by two-sided Student’s *t*-tests; other data in Figure 3 were analyzed by one-way ANOVA. * *p* < 0.05, ** *p* < 0.01, *** *p* < 0.001, **** *p* < 0.0001, ns, no significance.

According to the key time points of cardiac differentiation, indirect co-culture with hHSCs was performed with Transwell at different points on the 1st, 2nd, 7th, and 10th day when differentiation was turned on, and analyzed on the 3rd, 8th, and 15th day to explore the optimal time for co-culture of hHSCs with cardiac differentiation (Figure 3A). On the 9th day of differentiation, the hiPSC-CM, hiPSC-CM D7 + hHSC, and hiPSC-CM D10 + hHSC groups all showed cellular beating, and the beat rates steadily increased with time, while the hiPSC-CM D1 + hHSC and hiPSC-CM D2 + hHSC groups never showed beating (Figure 3B). Cells collected at different time points were lysed and assayed for myocardial enzymes. The results show that the levels of myocardial enzymes, creatine kinase MB isoenzyme (CK-MB) (Figure 3C), lactate dehydrogenase (LDH) (Figure 3D), and myoglobin (MYO) (Figure 3E) were much higher in the hiPSC-CM D7 + hHSC group than those in the other groups. The expressions of cardiac marker genes, GATA binding protein 4 (GATA4), and NK2 homeobox 5 (NKX2.5) identified by qPCR were likewise consistent with ELISA results (Supplementary Figure S5D). In addition, we detected the differentiation rate by flow cytometry and found that the proportion of cTNI-expressing cells in the hiPSC-CM D7 + hHSC group was 64.7%, which was considerably greater than the other groups, even though the rate of change did not differ statistically (Figure 3F). Then, we detected the representative genes at key time points of cardiac differentiation by qPCR, and their expressions followed the same trend as the myocardial enzymes (Figure 3G–I). All these results confirmed that the 7th day, which was the cardiac progenitor cell stage, was the optimal time for co-culture.

### 2.4. Comparison of Structural and Functional Features of Co-Cultured and Independently Induced CMs on MatriClone and MatriClone-Plastic

After determining the concentration of MatriClone, the type of microcarrier, and the optimal time point for co-culture, we performed cardiac differentiation on MatriClone and MatriClone-Plastic, respectively, and co-cultured them with hHSCs on the 7th day of differentiation to better understand the advantages of co-culture on cardiac development (Supplementary Figure S6A). We also did not apply hiPSC-CM enrichment methods [26] at the end of the cardiac differentiation protocol. Flow cytometry findings revealed that the co-cultured induced CM groups (MatriClone + hHSC and MatriClone-Plastic + hHSC) had a larger proportion of cTNI-expressing cells than the independently induced CM groups (MatriClone and MatriClone-Plastic) (Supplementary Figure S6B). Then, we collected cells according to the key time points of cardiac differentiation. The myocardial enzyme assay showed that the levels of CK-MB, LDH, and MYO (Supplementary Figure S6C–E) were significantly higher in the co-culture induced CM groups than in the independently induced CM groups. However, there was no difference between the MatriClone and MatriClone-Plastic groups, and similarly between the MatriClone + hHSC and MatriClone-Plastic + hHSC groups. The qPCR results showed that the expression of undifferentiated cell marker genes in the four comparison groups was significantly reduced following the start of differentiation, with almost no expression seen on the 15th day (Supplementary Figure S6F). On the 3rd, 8th, and 15th days of differentiation, the expressions of mesoderm marker genes (Supplementary Figure S6G), cardiac progenitor marker genes (Supplementary Figure S6H), and cardiac marker genes (Supplementary Figure S6I,J) followed the same trend as the myocardial enzymes. Similarly, there was no difference between the MatriClone and MatriClone-Plastic groups, nor between the MatriClone + hHSC and MatriClone-Plastic + hHSC groups.

**Figure 4:**
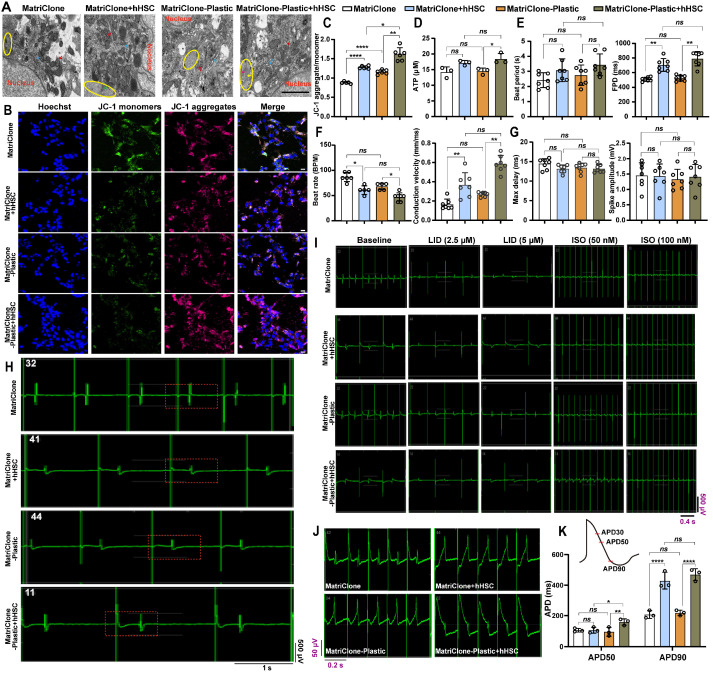
Comparison of structure and function of co-cultured and independently induced CMs. (**A**) Representative electroscopic images of CMs in each group. Red arrows indicate mitochondria, blue arrows indicate sarcoplasmic reticulum, yellow circles indicate myofilaments, the green arrow indicates the Z line, the yellow arrow indicates the H line, and the magenta arrow indicates the intercalated disk. Symbols (*) indicate lipid droplets; scale bars = 2 μm. (**B**) Representative images of JC-1 staining of hiPSC-CMs of the four comparison groups on the 15th day. The MMP of living hiPSC-CMs was detected by JC-1 fluorescent probe; scale bars = 25 μm. (**C**) The fluorescence intensity of JC-1 staining in (**B**) was quantified by Image J 1.54p (*n* = 5–6). (**D**) The ATP levels of hiPSC-CMs in each group were detected by ATP assay kit (*n* = 3). (**E**) The differences in beat period and FPD in cultured hiPSC-CMs of the four comparison groups were detected by MEA (*n* = 7). (**F**) The differences in beat rate and conduction velocity in cultured hiPSC-CMs of the four comparison groups were detected by MEA (*n* = 5–7). (**G**) The differences in max delay and spike amplitude in cultured hiPSC-CMs of the four comparison groups were detected by MEA (*n* = 6–7). (**H**) Representative continuous waveform of one electrode on a single well by MEA, and a waveform is displayed within the red square. Spontaneous beating hiPSC-CMs were confirmed by the extracellular FP recorded on the 6th day after cell seeding. (**I**) Representative FP signals from a single well after added LID and ISO in order to detect the differences in FPD during depolarization/repolarization of hiPSC-CMs in the four comparison groups. (**J**) Representative LEAP signals from a single well of hiPSC-CMs in the four comparison groups. (**K**) The quantification of APD50 and APD90 in (**J**) by Image J 1.54p (*n* = 3). All data were expressed as mean ± SD. Data in (**C**,**E**,**G**,**K**)were analyzed by one-way ANOVA; data in (**D**,**F**) were analyzed by nonparametric testing. * *p* < 0.05, ** *p* < 0.01, **** *p* < 0.0001, ns, no significance.

To further understand the structure of CMs, we fixed the amount of hiPSC-CMs in each group and detected them by transmission electron microscopy (TEM). Compared to independently induced CMs, co-culture induced CMs had more obvious aggregates of mitochondria, with full morphology and much higher length–width ratios. Meanwhile, co-cultured induced CMs exhibited CM-specific features, such as the Z-band, H-band, and intercalated disk (Figure 4A and Figure S7A). Bridging integrator 1 (BIN1) is also a classic structural marker of CMs, which is often used to evaluate their maturity. Our fluorescence staining results are also similar to those from the TEM (Supplementary Figure S7B,C). To assess mitochondrial function, we measured the mitochondrial membrane potential (MMP) and the ATP levels of hiPSC-CMs in each group. Co-culture induced CMs had higher MMP than independently induced CMs. We also found that the level of ATP of hiPSC-CMs in the MatriClone-Plastic + hHSC group was significantly higher than that in the MatriClone-Plastic group (Figure 4B–D).

The electrophysiological function of CMs was performed on the multi-electrode assay (MEA) system, which allows simultaneous label-free measurements of extracellular FP from CMs in multiple wells, facilitating their use in measuring real-time CM excitability in culture plates or for monitoring long term cardiac maturation [28,29]. Compared to conventional patch-clamp techniques, which are technically demanding, low-throughput, and require invasive penetration of the cell membrane, both local extracellular action potential (LEAP) and MEA enable non-invasive, high-throughput recordings of electrophysiological activity from multiple CMs simultaneously. MEA is particularly suited for long-term dynamic monitoring of population-level beating activity, while LEAP can achieve optical recording of single-cell action potentials with higher spatial resolution. hiPSC-CMs on the 15th day from each group were re-seeded onto MEA plates, and data were collected after restoring regular beats for 6 days of culture. Analysis of the electrical signals from each group found that, compared to the independently induced CM groups, there were substantial increases in field potential duration (FPD) and conduction velocity and a decrease in beat rate in the co-culture induced CM groups (Figure 4E,F). Other characteristics, such as beat period, max delay, and spike amplitude, showed no statistically significant difference (Figure 4G). Continuous waveforms showed the contractility of hiPSC-CMs on one electrode of the well (Figure 4H), and the heatmap showed the conduction signal of CM monolayers in each group (Supplementary Figure S7D). We further examined the electrophysiologic functions of hiPSC-CMs in each group after treatment with 5/2.5 μM lidocaine (LID) or 100/50 nM isoproterenol (ISO). Following treatment, the hiPSC-CMs in each group had regular depolarization and repolarization shifts (Figure 4I and Figure S7E,F).

The FP derives from the underlying cardiac action potential (AP) [30]; hence, we applied electrical signals to the planar microelectrodes to induce AP signals by the LEAP assay of the MEA system. Following induction, the signal known as LEAP had substantially increased in amplitude and changed in shape to resemble the cardiac AP (Figure 4J). We quantified the automated detection of APD50 and APD90 from the LEAP signals, and found that the values of APD90 in the co-culture induced CM groups were significantly higher than those in the independently induced CM groups (Figure 4K). At the same time, we used another hiPSC cell line B1 (derived from human blood cells) and co-cultured it with another hHSC line (derived from human cord blood) on the 7th day of cardiac differentiation. We repeated some of the above experiments and got similar results (Supplementary Figure S8).

In summary, co-culture induced CMs were found to exhibit more mature and stable structure and electrophysiological functions.

### 2.5. RNA-Seq Revealed the Mechanism of Cardiac Differentiation Co-Cultured with hHSCs on MatriClone and MatriClone-Plastic

**Figure 5:**
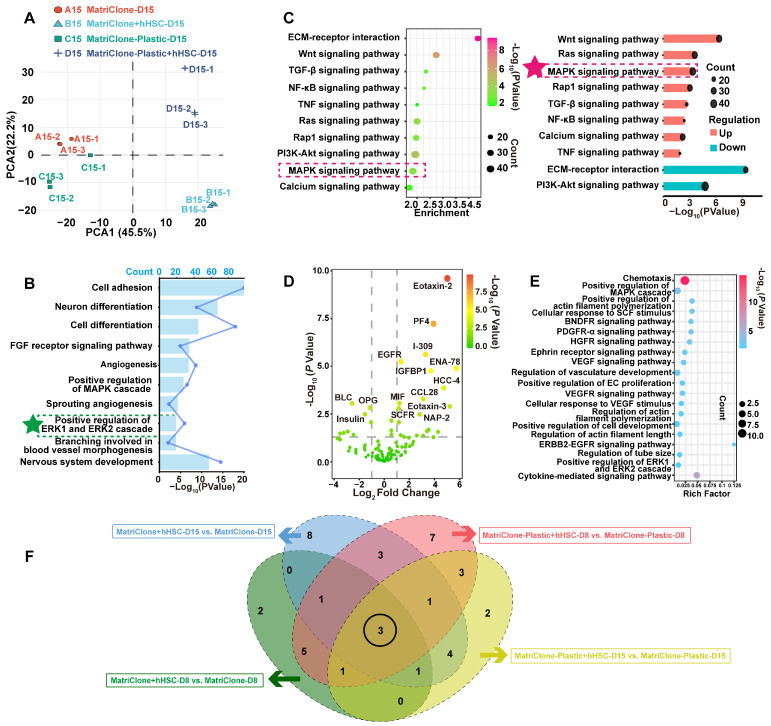
Comparison of gene expression profiles in hiPSC-CMs by RNA-seq and cytokine-related proteins in supernatants by cytokine assay from the MatriClone-Plastic group and the MatriClone-Plastic + hHSC group on the 15th day. (**A**) PCA was performed based on the common genes of MatriClone-D15, MatriClone + hHSC-D15, MatriClone-Plastic-D15, and MatriClone-Plastic + hHSC-D15. (**B**) BPs in GO enrichment analysis of DEGs between MatriClone-Plastic + hHSC-D15 and MatriClone-Plastic-D15; BPs marked with green asterisks and dashed lines were significantly enriched in both comparisons (“MatriClone + hHSC vs. MatriClone” and “MatriClone-Plastic + hHSC vs. MatriClone-Plastic”). (**C**) KEGG enrichment analysis of DEGs between MatriClone-Plastic + hHSC-D15 and MatriClone-Plastic-D15. The KEGG pathways marked with magenta asterisks and dashed lines overlapped across the four comparison groups and played a role in the upregulation process. (**D**) Volcano plots for of the cytokine assay in supernatants of MatriClone-Plastic + hHSC-D15 and MatriClone-Plastic-D15. (**E**) BPs in GO enrichment analysis of DEPs between MatriClone-Plastic + hHSC-D15 and MatriClone-Plastic-D15. (**F**) Venn diagrams of DEPs in the four comparison groups.

According to our earlier findings, the difference in marker gene expression in the co-cultured and independently induced CMs widened from the 8th day on both MatriClone and MatriClone-Plastic, suggesting that hHSCs could indeed promote cardiac development and maturation. To determine if MatriClone and MatriClone-Plastic had different effects on cardiac differentiation, we used RNA-Seq to analyze the gene expression profiles of hiPSC-CMs on the 8th and 15th days. Principal component analysis (PCA) and cluster analysis (Figure 5A, Figure S9A and Figure S10A,B) revealed that the gene expression was similar in the independently induced CM groups on both the 8th and 15th day. The gene expression in the co-culture induced CM groups followed the same pattern as those in the independently induced CM groups. All these indicated that MatriClone-Plastic may replace MatriClone and produce large quantities of structurally and functionally more mature CMs.

To investigate the mechanism of hHSCs in promoting cardiac maturation, we used gene annotation and functional databases to perform gene ontology (GO) analysis of differentially expressed genes (DEGs) between the MatriClone + hHSC and MatriClone groups, and between the MatriClone-Plastic + hHSC and MatriClone-Plastic groups on the 8th and 15th day. According to GO analysis, the only overlapping biological process (BP) of “MatriClone + hHSC vs. MatriClone” and “MatriClone-Plastic + hHSC vs. MatriClone-Plastic” was cell differentiation, both on the 8th and 15th day. Meanwhile, the overlapping BPs also included angiogenesis, sprouting angiogenesis, the positive regulation of the ERK1 and ERK2 cascade, and the positive regulation of the MAPK cascade on the 15th day. In addition, we also found that, compared to “MatriClone + hHSC-D15 vs. MatriClone-D15”, there were some BPs related to neural development, such as neuron differentiation and neuron system development (Figure 5B, Figure S9B and Figure S10C,D). These findings suggested that hHSCs could promote cardiac maturation by regulating these BPs.

To identify the key signaling pathways associated with these DEGs, pathway analysis was conducted via the Kyoto Encyclopedia of Genes and Genomes (KEGG) database. Among the common uni-genes of KEGG analysis, the MAPK signaling pathway, involved in cell growth and differentiation, was the only one that overlapped across groups and played a role in up-regulation (Figure 5C, Figure S9C and Figure S10E,F). ERK has been shown to be involved in the MAPK signaling pathway. Studies have confirmed that the MAPK/ERK signaling pathway has a crucial role in cell biological responses such as cell proliferation, differentiation, and transformation by transducing extracellular stimulatory signals into cells and their nuclei [31].

Based on these findings, we inferred that hHSCs promote cardiac maturation through the MAPK/ERK pathway.

### 2.6. Cytokine Assay Reveals That Activation of the EGFR by Cytokines Secreted by hHSCs Promoted Cardiac Differentiation and Maturation

In our study, hHSCs were indirectly cultivated with cardiac differentiation via Transwell to mimic the simultaneous development of hematopoietic and cardiac lineages during embryonic development, which interaction was mediated by cytokines. To clarify the most efficacious cytokines and pathways, we performed cytokine assay analysis. Based on RNA-seq time points, we screened the differentially expressed proteins (DEPs) of the four comparison groups (|log_2_fold change| ≥ 0.263, *p* value < 0.05) and found that the cytokines changed dynamically at different time points (Figure 5D, Figure S9D and Figure S10G,H). GO analysis was performed on the DEPs of the four comparison groups, and found the follow overlapping BPs of the “MatriClone + HSC group vs. MatriClone group” and the “MatriClone-Plastic + HSC group vs. MatriClone-Plastic group” at the 8th and 15th day: positive regulation of MAPK cascade, regulation of ERK1 and ERK2 cascade, and regulation of the ERBB2-EGFR signaling pathway (Figure 5E, Supplementary Figures S9E and S10I,J). All these results indicated that hHSCs facilitated cardiac development and maturation through multiple mechanisms.

To identify specific cytokines, we constructed Venn diagrams of significantly altered protein sets in the four comparison groups (Figure 5F), and further identified three overlapping DEPs, namely immunoglobulin binding protein 1(IGBP1), stem cell factor receptor (SCFR), and the EGFR, whose expression was confirmed in cell supernatants (Supplementary Figure S10K). The EGFR, an upstream signal of the MAPK/ERK pathway, plays a crucial role in placental and embryonic development and can be directly detected as a soluble receptor in healthy placentas and human biological fluids [32,33]. As a result, the EGFR was selected as a focal factor for our following mechanistic research.

### 2.7. hHSCs Secreted a Variety of Cytokines and Activated the EGFR/MAPK/ERK Signaling Pathway to Promote Cardiac Differentiation, Resulting in Structurally and Functionally Improved CMs

**Figure 6:**
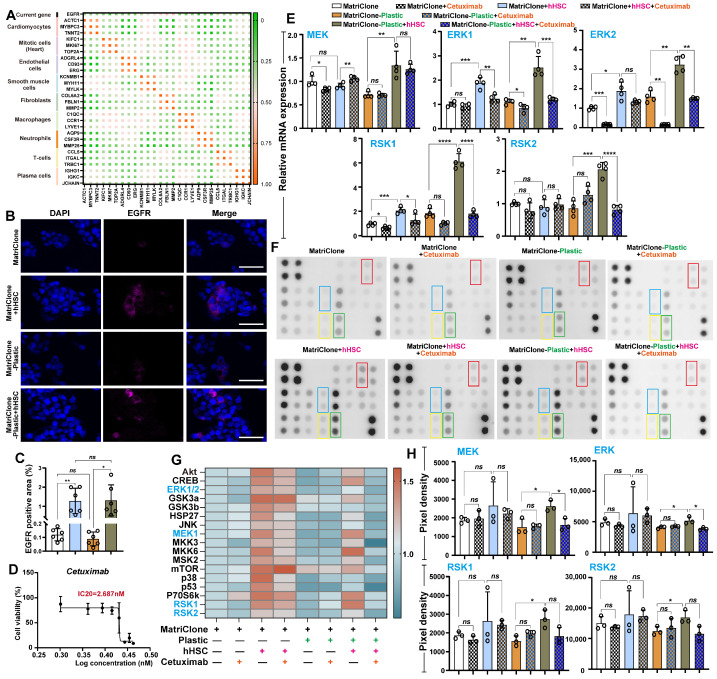
hHSCs activate the EGFR/MAPK/ERK signaling pathway to promote cardiac development and maturation. (**A**) Analysis of EGFR gene expression was acquired from single cell types in the HPA database. (**B**) Representative immunofluorescence images of EGFR expression in hiPSC-CMs of the four comparison groups on the 15th day; scale bars = 50 μm. (**C**) The EGFR-positive area per field in (**B**) was quantified by Image J 1.54p (*n* = 6). (**D**) The IC20 of Cetuximab was determined using the Cell Counting Kit-8 (CCK8) assay (*n* = 3). (**E**) qPCR analysis was used to characterize MAPK pathway indicators MEK, ERK1/2, and RSK1/2 in hiPSC-CMs on the 15th day in each group (*n* = 4). (**F**) Representative images of the MAPK phosphorylation assay of hiPSC-CMs in each group scanned with a chemiluminescent imaging system. Dot blots are shown: red boxes indicate ERK, blue boxes indicate MEK, yellow boxes indicate RSK1, and green boxes indicate RSK2. (**G**) Heatmap of MAPK phosphorylation assay performed with hiPSC-CMs in each group. (**H**) Quantitative results of MEK, ERK, and RSK1/2 of the original blot (*n* = 3). All data were expressed as mean ± SD. Normality testing was performed using the Shapiro–Wilk test before selection of statistical tests as appropriate. Data in (**C**,**D**,**G**)were analyzed by two-sided Student’s *t*-tests. * *p* < 0.05, ** *p* < 0.01, *** *p* < 0.001, **** *p* < 0.0001, ns, no significance.

Since there are no published studies on the relationship between the EGFR and cardiac development, single-cell sequencing data from the HPA database (https://www.proteinatlas.org/, accessed on 5 April 2025) were searched to focus on the relationship between the EGFR and CMs. Heatmap analysis revealed that the EGFR was strongly expressed in CMs and mitotic cells of the heart (Figure 6A). Immunofluorescence staining showed that the EGFR was more highly expressed in co-cultured induced CMs than in independently induced CMs on the 15th day (Figure 6B,C). Studies have shown that activated EGFR transmits extracellular signals primarily by activating the downstream ERK1/2 and protein kinase B (Akt) pathways [30,34]. Combined with the up-regulation of the EGFR in cell supernatants, we suggested that the EGFR participated in cardiac development and maturation and activation of the MAPK/ERK signaling pathway.

Taken together, we assumed that multiple cytokines secreted by hHSCs activated the EGFR and thus regulated the MAPK/ERK pathway to exert a pro-cardiac developmental effect. To confirm this assumption, we added the EGFR inhibitor Cetuximab (concentration: 2.687 nM) (Figure 6D) on the 7th day of cardiac differentiation and cultured it until the 15th day, amounting to 8 days of co-culture with Cetuximab, to assess the effect of EGFR inhibition on the MAPK/ERK signaling pathway at the protein and gene level. MAPK phosphorylation assay was used to determine the phosphorylation levels of major MAPK signaling pathway indicators. The results showed that, when compared to independently induced CMs, the expression of MAPK/ERK pathway indicators (MEK, ERK1/2, RSK1/2) was differentially increased in co-cultured induced CMs, but this positive effect was reversed after the addition of Cetuximab, especially in the MatriClone-Plastic + hHSC group (Figure 6E–H). This indicated that hHSCs played a critical role in cardiac development by activating the EGFR/MAPK/ERK signaling pathway.

## 3. Discussion

hiPSC-CM-based transplantation therapies have emerged as an alternative option for repairing damaged myocardium and have a wide range of potential applications. Although cardiac differentiation protocols have been continuously optimized, the current commonly used protocols have been performed in planar, with limited scalability, low yields, and large inter-batch and inter-well variations. Studies have begun to focus on microcarrier-based suspension culture systems, but the lack of standardized differentiation protocols, the animal-derived components in the culture system, and the immature functional properties of resulting cells present substantial practical barriers to reproducible studies using hiPSC-CMs [6,35]. Here, we optimized our laboratory’s cardiac differentiation protocol [25] in planar and proposed a cardiac differentiation protocol based on a xeno-free matrix-coated microcarrier, resulting in a high amount of CMs with CM properties and low inter-batch variation.

For CM modeling in vitro, the interaction and co-development of multiple progenitor cells during embryonic development synergistically promotes the maturation of CM morphology and function, making it difficult to adequately mimic the cellular microenvironment with single cell development [36]. Given the critical role of hematopoietic signaling in cardiac development, we co-cultured with hHSCs at the cardiac progenitor cell stage to obtain a large number of high-purity hiPSC-CMs, extensively characterized the co-culture induced CMs, and demonstrated that they had robust transcriptional, structural, metabolic, and functional properties of hiPSC-CMs. To our knowledge, this is the first work to integrate hHSCs with cardiac differentiation on a xeno-free matrix and microcarrier to generate highly matured hiPSC-CMs in vitro. This co-culture cardiac differentiation protocol and the subsequent in-depth morphological and functional characterization of co-culture induced CMs provides a new and promising basis for hiPSC-CM application and co-culture bioreactor research for future disease modeling and therapeutic CM replacement.

Previous research used bioreactors to convert suspended hiPSCs into embryoid bodies (EBs), which were then differentiated into CMs, resulting in large-scale cardiac production [6,37,38]. However, there is a self-differentiation process in EBs: it is hard to control its size and state, and it has a greater impact on cell differentiation in vitro, making standardization harder. Microcarriers are small beads with diameters ranging from 100 to 300 μm that have a high surface-to-volume ratio and may bind to anchorage-dependent cells to form microcarrier–cell complexes [39]. They can be used to scale up cell production in rotating flasks and bioreactors using only a minimal amount of culture medium, which has clear technical benefits [40]. However, cell and organ functions rely highly on intercellular communication and cell–ECM interactions. While microcarriers have mechanical properties similar to ECMs, they cannot duplicate the intricate structural and compositional characteristics of actual cells and organs [41,42]. To enhance hiPSC adherence, a matrix should be placed on the microcarrier surface [43]. We conducted comprehensive screening studies to identify Good Manufacturing Practice (GMP)-compliant CMs in xeno-free settings. Previous studies have shown that natural ECM or synthetic recombinant proteins can effectively support the adhesion, self-renewal, and maintenance of pluripotency in pluripotent stem cells, including fibronectin, laminin, and its various subtypes [44,45,46]. Compared to the commonly used Matrigel, these bioactive substances offer advantages such as xeno-free components, well-defined chemical composition, and simplified handling. To address the above issues, our study performed comprehensive screening experiments aimed at identifying the matrix that complies with GMP standards and is free of animal-derived components. Matrigel is derived from mouse sarcoma tissue and consists primarily of laminin [47]. Previous studies have reported that human recombinant laminin 511 can support the long-term culture of hESCs in vitro [45]. Therefore, after extensive screening experiments, we selected MatriClone consisting of recombinant laminin 511 E8 fragments. Compared to other laminin isoforms, it exhibits stronger adhesion. In order to find the most suitable microcarrier for hiPSC culture, we searched for commercially available microcarriers capable of being coated with the matrix. From these, we selected four xeno-free microcarriers, which are the four types of microcarriers mentioned previously. After multiple experiments, we finally chose the microcarrier named “Plastic” for the subsequent research.

First, from the original cardiac differentiation protocol, bovine serum albumin was substituted for recombinant human serum albumin, which had the same effect, and was purer and more stable in batches and posed no risk of bloodborne virus infection. Subsequently, the commonly used Matrigel extracted from Engelbreth-Holm-Swarm (EHS) mouse tumors was replaced with commercialized MatriClone. Considering the cost, 0.25–1 μg/cm^2^ was the most suitable MatriClone density for hiPSC expansion and cardiac differentiation. Next, we evaluated commercially available xeno-free microcarriers and coated the surface with different densities of MatriClone, discovering that 1 μg/cm^2^ MatriClone-coated plastic was able to maintain hiPSC phenotype and cardiac differentiation for prolonged periods in vitro. In our studies with 24-well plates, the plastic concentration used was relatively low and the culture was initiated from a relatively low cell inoculum, which satisfactorily mimicked standard 2D differentiation in culture plates. Our culture procedure on 1 μg/cm^2^ MatriClone-coated plastic resulted in increased cell production and differentiation efficiency compared to planar cultures.

The use of stem cell-derived CMs for repairing damaged CMs provides a new therapeutic approach for fundamentally saving infarcted or failing hearts in clinical practice. However, the current CMs for transplantation have problems such as xenogeneic components, low maturity, and structural imperfections, which have become the key obstacles for the further promotion and application of stem cell-derived CMs. Therefore, the maturity of CMs differentiated in vitro has been a critical and challenging topic in cell therapy. At present, most studies promote the maturation of stem cell-derived CMs in vitro by chemical induction, physical stimulation, microRNA (miRNA) regulation, and other methods. Stem cells, especially ESCs and iPSCs, have a strong dependence on cytokines during the culture process, including basic fibroblast growth factor (bFGF), bone morphogenetic protein (BMP), transforming growth factor β (TGFβ), and Activin A [48,49,50]. Therefore, adding or removing certain cytokines during the differentiation process, which is low-cost and easy to operate, has also become the most widely used differentiation protocol at present. However, due to the influence of chemokine brand and batch effects, the differentiation efficiency and cell purity of this protocol are low, making it difficult to meet the demands of large-scale experiments. The process also tends to introduce xenogeneic components, which are difficult to deploy in clinical transplantation. The physical microenvironment of electromagnetic fields and mechanics can provide the necessary microenvironment for the development and survival of CMs. Therefore, in recent years, some studies have adopted physical methods such as adding mechanical stimulation and electrical stimulation to intervene in stem cell differentiation, simulate the microenvironment of CM growth, and promote the maturation of stem cell-derived CMs [51,52]. However, cardiac differentiation induced by physical stimulation is only applicable to specific stem cell lines. Moreover, the experimental process is cumbersome, and there is no fixed scheme for the screening and combination of the types, modes, and parameters of physical stimulation, which requires multidisciplinary cooperation and extensive experimental explorations. miRNA is a key regulator of cardiac development and function. Recent studies have shown that some miRNAs affect the cardiac proliferation and maturation axis and can regulate the development of cardiac structure and function [53,54,55]. Therefore, when inducing stem cells to differentiate into CMs, the regulation of specific miRNAs can promote the maturation of stem cell-derived CMs. However, the cost of differentiation is high, and there is a risk of viral contamination as well as a tendency to form teratomas.

In addition to the individual culture methods, the emergence of contact co-culture also provides a new option for the induction of differentiation. Direct or indirect co-culture with other cells can realize the exchange of information and communication between cells, which is closer to the environment in vivo [56]. Compared with direct co-culture, indirect co-culture does not involve direct contact between the two cells, thus isolating the influence of physical factors on the differentiation process. Indirect co-culture only retains the exchange of chemicals such as cytokines secreted between the cells and the effect of the ionic gradient, which facilitates the separation of cells and subsequent detection. The contact co-culture of stem cells and various kinds of somatic cells is the most common study. HSCs possess unique biological characteristics, including self-renewal, multi-lineage differentiation, apoptosis, rest, and trafficking, thereby participating in tissue repair and regeneration [57,58,59,60,61,62]. Studies have shown that the differentiated products of HSCs can secrete various cytokines, participating in the regulation of cardiac paracrine signaling and cellular communication. When co-cultured with CMs, they can drive the phenotypic polarization of CMs, enhance CM contractility, and promote the proliferation, maturation, and functional integration of CMs [19,20,21,22,23,24]. During embryonic stem cell development, the mesoderm is assumed to arise in two waves, with the first wave primarily promoting hematopoietic (formed by the directed differentiation of HSCs) and endothelial lineages, and the second wave promoting cardiac and endothelial lineages [63,64]. Further studies have demonstrated that the hematopoietic and cardiac lineages are antagonistic to each other during mesoderm formation [16,17]. Therefore, based on the above studies and due to the complexity of blood cell types, we co-cultured hHSCs, the origin cells of human blood cells, with cardiac differentiation to observe their relationship with cardiac development and maturation. In this study, we co-cultured the cells with hHSCs on the 1st and 2nd day of cardiac differentiation, a stage of mesodermal differentiation, and found that it did not promote their differentiation and even inhibited cardiac development, which supported this conclusion. To date, the critical time point for HSCs engaged in cardiac development has not been verified, so we added the time point of co-culture in the subsequent study. When hiPSCs were differentiated into cardiac progenitor cells and then co-cultured with hHSCs, the harvested hiPSC-CMs showed dramatically enhanced transcription and myocardial enzyme expression. Then, we performed cardiac differentiation on 1 μg/cm^2^ MatriClone-Plastic and co-cultured it with hHSCs at the cardiac progenitor cell stage. Unpurified hiPSC-CMs produced more mature structures (Z-band, H-band, and intercalated disk) and stable electrophysiological functions, including better mitochondrial structure and function. Thus, the differentiation rate and maturity of co-cultured induced CMs on 1 μg/cm^2^ MatriClone-Plastic were significantly higher than independently induced CMs on MatriClone.

Cardiac development is a complex process involving many molecular signals and pathways. In order to clarify the molecular mechanism of hHSCs on cardiac development, we performed RNA-seq on the cells on the 8th and 15th day in four comparison groups and found that it may be guided by the MAPK/ERK signaling pathway. Our further analysis of the RNA-seq data revealed that the positive regulation of the MAPK signaling pathway continued to increase over time, which further supports our hypothesis. The MAPK signaling pathway regulates a variety of cellular physiological processes, and ERK is a subtype of the MAPK signaling system. The ERK pathway can be activated by a variety of cytokines and follows the MAPK cascade effect to promote ERK1/ERK2 phosphorylation. Previous studies have shown that cytokines can mediate the growth, determination, and differentiation of the embryonic heart through intercellular signaling at various stages of cardiac development [9]. In this study, we found that the cytokines secreted by hHSCs at different time points changed dynamically by cytokine assay, driving cardiac growth and maturation. Several cytokines have been reported to promote human embryonic development [65,66], but no single factor has been reported to replace the functions of all others. Therefore, the combination of various cytokines is a potential way to improve the efficiency of cell maturation and embryonic development.

Cytokines work in a variety of ways, including binding to the appropriate receptors on the cell and activating intracellular signaling pathways. In our investigation, we discovered that the levels of the EGFR in the supernatant of co-cultured cells increased significantly throughout the procedure. The EGFR is a member of the human epidermal receptor (HER) family, also called HER-1, and widely expressed in a variety of cell types, presenting an intracellular tyrosine kinase (TK) activity and an extracellular structural domain (known as the soluble EGFR (sEGFR)) that can be shed and released into the bloodstream [32,67]. The EGFR induces cell proliferation and differentiation by activating its intracellularly located kinase pathway (ERK 1/2) through binding to ligands such as epithelial growth factor (EGF), transforming growth factor-alpha (TGF-α), and amphiregulin (AR) [68]. Through qPCR and the MAPK phosphorylation assay, we had more comprehensive evidence that hHSCs could effectively activate the EGFR, followed by inducing phosphorylation of ERK, which contributes to the increase in downstream ERK1/2 expression. After inhibiting the EGFR, the activation of ERK1/2 phosphorylation was limited and the expression of RSK1/2 was significantly decreased. It has been reported that the EGFR regulates cardiovascular structure and function and mediates a variety of cardiovascular diseases [68,69]. Studies have also shown that ERK is involved in the regulation of cardiac differentiation [70], and the sustained activation of ERK helps to induce the differentiation of human mesenchymal stem cells into CMs in vitro [71]. As a result, our study demonstrated that during the cardiac progenitor cell stage, hHSCs secrete a variety of cytokines that promote cardiac differentiation and maturation through activation of the EGFR/MAPK/ERK signaling pathway.

Compared with the existing cardiac differentiation protocols, the differentiation efficiency in this study is not considered high. This also indicates that the low efficiency and poor repeatability of manual operation have a significant impact on cardiac differentiation, so the microcarrier–bioreactor system is crucial for the clinical application of hiPSC-CMs. Moreover, the focus of this study is to optimize the existing protocol and construct a cardiac differentiation protocol based on xeno-free microcarriers, which will provide a research foundation for the future application of bioreactors. Although the xeno-free co-culture system in this study has not been subjected to extensive quantitative comparative analysis with the traditional differentiation process using animal-derived components, based on our research results and multiple stable cultures in vitro, and through a horizontal comparison of relevant literature studies, it can be determined that the xeno-free culture system of hiPSCs and hiPSC-CMs based on microcarriers proposed by us has good stability and scalability. Moreover, based on the absence of xeno-free components, it is easier for clinical translation and large-scale cultivation. Currently, no existing cardiac differentiation protocols achieve 100% efficiency, which means that the generation and proliferation of non-cardiomyocyte cells are inevitable during the differentiation process. Therefore, it is usually necessary to purify the differentiated cells in order to obtain the hiPSC-CMs desired. However, in our study, we did not apply hiPSC-CM enrichment methods after differentiation in order to directly compare the efficiency of cardiac differentiation across the different groups. In future studies, we will also select and purify iPSC-CMs through methods such as the lactate method [72] and p53 activation [73], followed by transplantation.

The study had some limitations. In this study, the hiPSC-CMs co-cultured with hHSCs were not compared with the data on the genes of adult and fetal hearts. This is because hiPSC-CMs, through the differentiation protocol of our research group in previous studies, have already been compared with data on the genes of adult and fetal hearts, confirming that this is highly consistent with late development of a fetal heart [25]. After treatment with Cetuximab, we did observe a moderate decrease in the expression of MEK. However, comparisons between certain groups did not show a statistically significant reduction. We consider that, on the one hand, our sample size is relatively small, and on the other hand, the effect of Cetuximab on MEK may actually be dose-dependent. Considering the above issues, we will design our future experiments with greater rigor, identify EGFR inhibitors that are more suitable for MEK, and further validate and substantiate our conclusions. Although this study has indicated that hHSCs might activate the EGFR by secreting certain factors and initiating downstream pathways, the specific cytokines that play a key role have not been identified. Therefore, in our future research, in order to reduce the use of hHSCs and save the cost of cultivation, we will expand the scope of our search to identify the key cytokines capable of upregulating the EGFR/MAPK/ERK pathway as alternatives to hHSCs. Due to financial and time restrictions, we did not perform the culture of hiPSCs and cardiac differentiation on MatriClone-Plastic in a bioreactor or stirred rotary flask, nor did we find a suitable bioreactor for co-culture. Based on the fact that our study concerned co-culturing cardiac differentiation with hHSCs, developing a bioreactor suitable for the co-culturing of both to yield large quantities of xeno-free, more functionally and structurally mature CMs will be the focus of our future work.

Overall, we have provided a xeno-free, scalable, and efficient hiPSC-CM expansion method for large-scale production, with numerous applications in drug metabolism, toxicity testing, and new drug discovery. Furthermore, our study has provided important scientific data for the development and implementation of co-culture bioreactors.

## 4. Materials and Methods

### 4.1. Cell Culture

hiPSC lines U1 and B1 (20–30 passages) were purchased from Beijing Saibei Biotechnology Co., Ltd. (Beijing, China). The undifferentiated hiPSCs were cultured on qualified Matrigel™ (Corning Incorporated, Corning, NY, USA)-coated 6-well plates (Corning Incorporated) in mTeSR medium (Stemcell Technologies, Vancouver, BC, Canada) in a humidified incubator at 37 °C and 5% CO_2_. The medium was refreshed daily and cultures were passaged at 80% confluence every 4–5 days. Cells were routinely passaged at a split ratio of 1:10 using the Gentle Cell Dissociation Reagent (GCDR) (Stemcell Technologies). hiPSCs were adapted to MatriClone (Advanced Instruments, Norwood, MA, USA)-coated plates for three passages prior to inoculation onto microcarriers. After verification, the numbers following the manufacturer’s name are the product numbers. According to the editors’ requirements, these do not fall under the necessary content that needs to be supplemented. Therefore, we have removed this content from this version.

Human Bone Marrow Derived HSCs (primary generation) were purchased from Beijing ZEPING Bioscience & Technology Co., Ltd. (Beijing, China), and cultured with X-VIVO 15 Serum-free HSC medium (Lonza, Basel, Switzerland). Human Cord Blood Derived HSCs (primary generation) were provided by Xiamen Immocell Biotechnology Co., Ltd. (Xiamen, China), and cultured with special medium for hHSCs (Xiamen Immocell Biotechnology Co., Ltd.). The two HSC lines were respectively suspended cultured in low-attachment 6-well plates (Corning Incorporated) with the corresponding medium in a humidified incubator at 37 °C and 5% CO_2_. The medium was replaced every 2–3 days, and the cell density was maintained at 80–90% to ensure optimal conditions for the subsequent passaging culture.

### 4.2. MatriClone and Microcarriers

MatriClone is a purified recombinant soluble laminin fragment designed to support the early growth of isolated iPSCs [74]. Dilute MatriClone in DPBS (Gibco, Waltham, MA, USA) and incubate for 1 h at 37 °C to coat the plates.

In order to select a xeno-free microcarrier suitable for hiPSC culture, we purchased the SoloHill^®^ Microcarrier Starter Kit (Sartorius, Göttingen, Germany) containing the following four microcarriers without animal components: Hillex, Plastic Plus, Plastic, and Star-Plus (Supplementary Table S1). Dry microcarrier material was pretreated for cell culture. Briefly, the four microcarriers, with 360 cm^2^/g of superficial area, were mixed thoroughly with deionized water to prepare the microcarrier stock solution and stored at 4 °C after sterilization by autoclaving. Coating of microcarriers with diluted MatriClone at 1 μg/cm^2^ was carried out for 2 h at room temperature (RT). Prior to cell inoculation, microcarriers were incubated for 30 min at 37 °C in mTeSR medium supplemented with 10 μM of ROCK inhibitor Y-27632 (Med Chem Express, Monmouth Junction, NJ, USA).

### 4.3. Long-Term Cultivation of hiPSCs on Microcarriers and Purity Analysis

hiPSCs were seeded onto the four microcarriers coated with MatriClone at 1 μg/cm^2^ (MatriClone-microcarriers), as described previously [75,76]. We used low-attachment 24-well plates (Corning Incorporated) with 3 cm^2^ of microcarrier superficial area per well, and cells were inoculated at an initial density of 5 × 10^4^ cells/cm^2^. The medium was changed daily and the cells were harvested when the culture reached confluence. hiPSCs adherent on MatriClone-microcarriers were washed once in DPBS and treated with GCDR for 4 min at RT before being aborted with mTeSR medium containing Y27632. Then, the mixture was filtered through 40 μm cell strainers (Yeasen Biotechnology, Shanghai, China) to remove the microcarriers and seed onto freshly prepared MatriClone-microcarriers. hiPSCs were continuously cultured on MatriClone-microcarriers and analyzed for pluripotency after the 10th passaging.

### 4.4. hiPSC Differentiation Potential In Vitro

The Stemdiff^TM^ Trilineage Differentiation Kit (Stemcell Technologies) provides functional verification of the ability of hiPSC lines to differentiate to the three germ layers—ectoderm, mesoderm, and endoderm—and therefore we used it to direct the differentiation of long-term cultivation of hiPSCs on MatriClone and MatriClone-microcarriers to all three germ layers to assess differentiation potential. The protocol was performed according to the manufacturer’s instructions (Supplementary Figure S1). Cells were harvested and/or fixed on the 5th day of mesodermal and endodermal differentiation and on the 7th day of ectodermal differentiation to analyze each lineage-specific marker.

### 4.5. Saponin^+^ Compound Preparation

The saponin^+^ compound, derived from traditional Chinese medicine, is mainly composed of lignocaine, salvinorin B, sphingosine-1-phosphate, astragaloside, ginsenoside rg1, PLGF-2, a new isomer (scropolioside D), and other small molecules. This is a patented product developed in our own laboratory (PCT Patent: NO.US 12194063 B2), which is mainly used to promote cardiac differentiation and maturation in our protocol.

### 4.6. Generation of hiPSC-CMs in 2D Monolayer Culture

Monolayer cardiac differentiation was induced as previously described [25], with some modifications (Figure 1E).

The chemically defined medium used for cardiac differentiation included RPMI 1640 (Gibco), supplemented with Recombinant Human Serum Albumin (Oryzogen, Wuhan, China), MEM Non-Essential Amino Acids Solution (100×) (Gibco), Ascorbic Acid 2-Phosphate Magnesium (Sigma-Aldrich, St. Louis, MO, USA), and 1-Thioglycerol (Sigma-Aldrich), hereafter referred to as cardiac differentiation medium (CDM).

Briefly, hiPSCs were washed once with DPBS, detached by incubation with Accutase (Stemcell Technologies) for 2 min, and seeded onto 24-well plates (Thermo Scientific, Waltham, MA, USA) pretreated with 1 μg/cm^2^ MatriClone at a density of 2.23 × 10^5^ cells/cm^2^ in mTeSR medium supplemented with 10 μM Y-27632.

The key steps in the entire differentiation process include the Mesoderm, Cardiac Mesoderm, Cardiac Progenitors, and the maturation of CMs. After 4 days of cell expansion culture, hiPSCs were treated with CDM containing 10 μM CHIR99021 (Sigma-Aldrich) for 24 h, followed by CDM containing 5 ng/mL bFGF (Peprotech, 100-18B, Cranbury, NJ, USA) for another 24 h. The cells were then treated with CDM containing the saponin^+^ compound for 24 h, thereby differentiating to the Mesoderm. At this point, half of the medium was replaced with CDM containing 5 μM IWP2 (Sigma-Aldrich) and the saponin+ compound, and the cells were cultured for two days to reach the Cardiac Mesoderm. On the 6th day of differentiation, cells were cultured in RPMI1640/3% KnockOut™ SR (Gibco) supplemented with the saponin+ compound, and the cells reached the Cardiac Progenitor stage after two days of culture. The same medium was continued, and was changed every two days until day 15.

We purified and enriched the cardiomyocytes on the 10th, 12th, and 14th days of cardiac differentiation using the lactate method [72]. In brief, we replaced the culture medium with glucose-free DMEM (Invitrogen, Carlsbad, CA, USA) containing 4 mmol/L L-lactate (Sigma-Aldrich) to screen and purify hiPSC-CMs through metabolic analysis.

### 4.7. Generation of hiPSC-CMs on MatriClone-Microcarriers

MatriClone-microcarrier cardiac differentiation was induced as previously described [25,77], with minor modifications (Supplementary Figure S3C). MatriClone-microcarriers were incubated for at least 30 min at 37 °C in mTeSR medium, followed by inoculation of cells in a low attachment 24-well plate at a starting cell concentration of 2.23 × 10^5^ cells/cm^2^. After 2 days of expansion culture, the cells reached confluency on the microcarrier surface. After washing the cells once with DPBS, the same cardiac differentiation protocol as described above was applied to the MatriClone-microcarriers. After 15 days of cardiac differentiation, hiPSC-CMs were dissociated by Cardiac-Specific Digestion Solution (Cellapybio, Beijing, China), filtered through 40 μm cell strainers to remove microcarriers, and prepared for downstream application or freezing.

### 4.8. Co-Culture of Cardiac Differentiation with hHSCs

Before co-culture on microcarriers, the final seeding density of hiPSCs was adjusted to 2.23 × 10^5^ cells/cm^2^. The final seeding density of hHSCs was adjusted to 2.15 × 10^4^ cells/cm^2^. These seeding densities were determined through optimization in our preliminary experiments, aiming to achieve optimal maturation without nutrient competition. The cell seeding ratio can be adjusted up or down from these values.

When exploring the optimal time for co-culture, we followed the experimental design shown in Figure 3A. Specifically, in the lower chamber of 0.4 μm Transwells (Corning, 3470), cardiac differentiation was induced as previously described. hHSCs in logarithmic growth phase were seeded at a density of 2.15 × 10^4^ cells/cm^2^ in the upper chamber of Transwells, and co-cultured with hiPSC-CMs at different time points on the 1st, 2nd, 7th, and 10th day of cardiac differentiation.

When performing the cell characterization experiments, we followed the experimental design shown in Supplementary Figure S6A. Specifically, on the 7th day of cardiac differentiation, when more than 80% of the cells reached the Cardiac Progenitor stage, hHSCs were collected and added to the microcarrier culture system at a seeding density of 2.15 × 10^4^ cells/cm^2^ for 8 days of co-culture.

Following their co-culture, the relevant culture conditions remained consistent with those used for the independent cultures, including the culture medium and the frequency of medium changes.

### 4.9. Immunofluorescence

Immunofluorescence of cells was performed in confocal dishes (Nest, Wuxi, China). Cells were fixed with 4% paraformaldehyde (Beyotime, Shanghai, China) for 30 min at RT, and permeabilized with 0.3% Triton X-100 (Sigma-Aldrich) for 20 min. After blocking with Normal Goat Serum (Zsgb-Bio, Beijing, China) for 1 h, primary antibodies were incubated overnight at 4 °C. Secondary antibodies were incubated for 1 h at RT in the dark. The nuclei were counterstained with DAPI Staining Solution (Beyotime), and after staining, the samples were washed four times with PBS for 5 min at RT each time.

All immunofluorescence images were captured using a confocal microscope (Olympus, Tokyo, Japan). A complete list of the primary and secondary antibodies used is provided Supplementary Table S2.

### 4.10. Flow Cytometry

Cells were dissociated into single cells with specific digestion solutions at 37 °C. After centrifugation, cells were fixed and permeabilized with eBioscience™ Foxp3/Transcription Factor Fixation/Permeabilization buffer (Thermo Scientific) for 1 h at RT, followed by centrifugation at 600× *g* for 5 min with permeabilization buffer (Thermo Scientific). Next, the cells were incubated with the directly labeled fluorescent antibodies for 30 min at RT in the dark. Analysis was performed using a FACSC Auto Flow Cytometer (LSRFortessa SORP, BD Biosciences, Bergen County, NJ, USA). All antibodies are listed Supplementary Table S3.

### 4.11. RNA Extraction and qPCR Analysis

Total RNA was extracted from cells using the AFTSpin Tissue/Cell Fast RNA Extraction Kit (ABclonal, Woburn, MA, USA). The RNA concentrations were measured using a Nanodrop 8000 Spectrophotometer (Thermo Scientific). The cDNA was synthesized in a T100 Thermal Cycler (Bio-Rad, Hercules, CA, USA) using ABScript Neo RT Master Mix for qPCR with gDNA Remover (ABclonal) according to the manufacturer’s protocol, and stored at −20 °C. qPCR was performed on the selected genes using the 2X Universal SYBR Green Fast qPCR Mix (ABclonal). qPCR assays were carried out in 96-well plates using a CFX96 Touch Real-Time PCR Detection System (Bio-Rad). Relative expression was calculated using the 2^(−ΔΔCT)^ method, and results are normalized to actin [6]. Supplementary Table S4 shows all primers used in this study.

### 4.12. Detection of Calcein/PI Staining

The Calcein/PI Cell Viability/Cytotoxicity Assay Kit (Beyotime) was used to detect the cell viability [78] of hiPSCs on MatriClone-microcarriers. The cultures were incubated with 1X Calcein AM and 1X PI for 30 min at 37 °C in the dark, and then staining effects were observed under a fluorescence microscope (Olympus). Calcein AM shows green fluorescence to label live cells, and PI shows red fluorescence to label dead cells.

### 4.13. Intracellular CK-MB, MYO, and LDH Assay

Intracellular CK-MB, MYO, and LDH production were detected by ELISA kits (Cloud-Clone Corp, Katy, TX, USA). Cells were collected at different time points on the 3rd, 8th, and 15th day of cardiac differentiation, and processed in accordance with the manufacturer’s instructions.

### 4.14. TEM

CMs collected from each group after centrifugation were added to the TEM Fixative (Servicebio, Wuhan, China) for 4 h at 4 °C. After washing with 0.1 M PB (pH 7.4), the cells were pre-embedded in 1% agarose solution for 2 h. The agarose blocks with cells were followed by fixation with 1% OsO_4_ in 0.1 M PB for 2 h at RT and washed with 0.1 M PB. Then, the cells were dehydrated with ethanol in increasing concentrations. Ultrathin sections were prepared after resin penetration and embedding and stained with 2% uranium acetate saturated alcohol solution and 2.6% lead citrate. Images were acquired by a HT7800 Transmission Electron Microscope (Hitachi, Tokyo, Japan).

### 4.15. MMP Assay

Cells were stained by the MMP Assay Kit with JC-1 (Beyotime). After digestion and re-inoculation of hiPSC-CMs on the 15th day from each group, 1X JC-1 staining solution was added and incubated at 37 °C for 20 min according to the instructions. Then, the staining working solution was removed, and the cells were washed twice with staining buffer and observed by the BX53 Fluorescence Microscope.

### 4.16. Intracellular ATP Level Measurement

The intracellular ATP level was determined by the ATP Assay Kit (Beyotime), according to the manufacturer’s guidelines. After lysing, the cells were centrifuged at 12,000× *g* for 5 min at 4 °C. The appropriate amount of ATP working solution was then added, and the entire solution was transferred to a 96-well white opaque plate (Beyotime) for chemiluminescence detection on a SpectraMax i3X Multi-mode Microplate Reader (Molecular Devices, Sunnyvale, CA, USA).

### 4.17. MEA Recordings

The MaestroEdge electrophysiology system (Axion BioSystems Inc., Atlanta, GA, USA) was used in this study. Briefly, CMs from each group were re-seeded onto a 24-well MEA plate (Axion BioSystems Inc., Atlanta, GA, USA) with 4 × 10^4^ cells/well according to the manufacturer’s protocol. After the cells returned regular beating, the MEA plates were transferred to the MEA device (37 °C and 5% CO_2_) and stabilized for 20 min before measurement. They were analyzed by the Cardiac Analysis Tool (Axion BioSystems Inc.). The representative MEA waveform includes the following main parameters for analysis: (1) Beat period refers to the interval between two peaks when the next depolarization occurs after depolarization. (2) FPD is defined as the time interval between peak depolarization and peak repolarization, similar to QT duration. (3) Conduction velocity represents the speed at which an electrical pulse propagates through heart tissue. (4) Max delay refers to the time difference in beat frequency detection between electrodes in a well. (5) Spike amplitude indirectly measures the amplitude of propagated action potentials of CMs from different groups. (6) APD30, APD50, APD90: Action potential duration from onset of excitation to 30%, 50%, and 90% of repolarization [28,79,80,81]. The parameter outputs of the MEA system used in our study, such as FPD, have been calibrated through the instrument’s standard preprocessing procedure. After correction via the Fridericia method for baseline drift and amplitude normalization, the results meet the requirements for subsequent analysis of CM electrophysiological activity and can be directly used for further statistical analysis.

### 4.18. Human Cytokine Assay

To define the cytokines secreted by hHSCs that play a role in pro-cardiac development and maturation, following our cardiac differentiation protocol, we collected cell supernatants from each group at different time points on the 8th and 15th day and assayed them using the Quantibody Human Cytokine Assay 2000 Kit (RayBiotech, Norcross, GA, USA) according to the manufacturer’s instructions. The fluorescence signals of the microarrays were visualized by the InnoScan 300 Microarray Scanner (Innopsys, Toulouse, France) and read by Mapix v1.0 software.

### 4.19. RNA-Seq Analysis

Total RNA was extracted from CMs using TRIzol^®^ Reagent (Invitrogen) according to the manufacturer’s instructions, and characterized by a NanoDrop 2000 Spectrophotometer (Thermo Fisher Scientific, Waltham, MA, USA) and an Agilent 5300 Bioanalyzer (Agilent Technologies, Santa Clara, CA, USA). Next, the mRNA was enriched using oligo (dT) magnetic beads and reverse transcribed to cDNA by a Superscript Double-Stranded cDNA Synthesis Kit (Invitrogen). Then, the sequencing library for each RNA sample was prepared by the Illumina Stranded mRNA Prep Ligation (Illumina, San Diego, CA, USA) according to the manufacturer’s instructions. After being quantified by Qubit 4.0, the sequencing libraries were performed on the NovaSeq X Plus platform (PE150, Illumina). The transcriptome data have been deposited into NCBI’s Sequence Read Archive (SRA) database under the accession number PRJNA1464369.

### 4.20. GO and KEGG Enrichment Analysis

The RNA-seq data were statistically analyzed by the Majorbio Cloud Platform (https://cloud.majorbio.com/page/tools/, accessed on 5 April 2025). |Log2 (Foldchange)| ≥ 1 and a *p* value < 0.05 were the thresholds for screening DEGs. GO and KEGG enrichment analysis were performed by The Database for Annotation, Visualization, and Integrated Discovery (DAVID (https://davidbioinformatics.nih.gov/, accessed on 6 April 2025). *p*-adjust (FDR-adjusted *p*-value using the Benjamini–Hochberg method) < 0.05 was considered significantly enriched. Enrichment results were imported into the SRplot online Platform (https://www.bioinformatics.com.cn/srplot, accessed on 7 April 2025) for graphing.

### 4.21. Phosphorylated Protein Assays (Phosphorylation Assay)

In order to validate the RNA-seq results, following our cardiac differentiation protocol, we collected cell lysates from each group at different time points on the 8th and 15th day and quantified protein concentration by BCA assay. Analysis was performed using a commercially available Human/Mouse MAPK Phosphorylation Assay (AAH-MAPK-1, RayBiotech, Norcross, GA, USA) according to the manufacturer’s instructions. Images were captured using a ImageQuant LAS4000 Scanner (GE Healthcare Corporate, Saint Louis, MO, USA), and the analysis software provided with the instrument was used to extract data for pre-analysis.

### 4.22. CCK8 Assay

The effect of Cetuximab on the viability of cardiomyocytes was detected using the CCK8 assay kit (Beyotime). After setting up a concentration gradient, the harvested cells were processed according to the manufacturer’s instructions.

### 4.23. Statistical Analysis

All data were expressed as mean values ± SD. Two-sided Student’s *t*-tests or one-way ANOVA was used for groups meeting the normality testing, and nonparametric testing was used for groups that did not meet the normality testing. Differences were considered statistically significant at a *p* value < 0.05. All experiments were conducted at least three times.

## Data Availability

Data are contained within the article and Supplementary Material, and materials and data supporting this study’s findings are available from the first or corresponding authors upon reasonable request. The transcriptome data have been deposited into NCBI’s SRA database under the accession number PRJNA1464369.

## Supplementary Materials

The following supporting information can be downloaded at: https://www.mdpi.com/article/10.3390/ph19060964/s1, Figure S1: Differentiation and identification of all three germ layers in MatriClone at different densities; Figure S2: Pluripotency identification of hiPSC-CMs in MatriClone at different densities; Figure S3: Cardiac differentiation on 1 μg/cm^2^ MatriClone-Plastic; Figure S4: Expansion and cardiac differentiation of hiPSC lines from B1 (derived from blood cells) on 1 μg/cm^2^ MatriClone-Plastic; Figure S5: hHSC identification; Figure S6: Comparison of characterization and structure of co-cultured and independently induced CMs; Figure S7: Comparison of structure and function of co-cultured and independently induced CMs; Figure S8: Co-culture of hiPSC line of B1 (derived from blood cells) and Human Cord Blood Derived HSCs for cardiac differentiation; Figure S9: Comparison of gene expression profiles in hiPSC-CMs by RNA-seq and cytokine-related proteins in supernatants by cytokine assay from the MatriClone group and the MatriClone + hHSC group on the 15th day. Figure S10: Comparison of gene expression profiles by RNA-seq and cytokine-related proteins in supernatants by cytokine assay of co-cultured and independently induced CMs on the 8th day. Table S1: The chemical characterization of four microcarriers; Table S2: Antibodies used in immunofluorescence analyses; Table S3: Antibodies used in flow cytometry; Table S4: Quantitative PCR primer list; Table S5: Cell lines. Video S1: hiPSC-CM beating on Matrigel; Video S2: hiPSC-CM beating on 0.25 μg/cm^2^ MatriClone; Video S3: hiPSC-CM beating on 0.5 μg/cm^2^ MatriClone; Video S4: hiPSC-CM beating on 0.75 μg/cm^2^ MatriClone; Video S5: hiPSC-CM beating on 1 μg/cm^2^ MatriClone; Video S6: hiPSC-CM beating on 1 μg/cm^2^ MatriClone-Plastic.

## Abbreviations

The following abbreviations are used in this manuscript:

hiPSCs	Human induced pluripotent stem cells
CMs	Cardiomyocytes
hiPSC-CMs	hiPSC-derived CMs
hHSCs	Human hematopoietic stem cells
EGFR	Epidermal growth factor receptor
MAPK	Mitogen-activated protein kinase
ERK	Extracellular signal-regulated kinase
cTNT	Cardiac Troponin T
cTNI	Cardiac Troponin I
OCT4	Octamer-binding transcription factor 4
SSEA4	Stage-specific embryonic antigen-4
CK-MB	Creatine kinase MB isoenzyme
LDH	Lactate dehydrogenase (LDH)
MYO	Myoglobin
GATA4	GATA binding protein 4
NKX2.5	NK2 homeobox 5
