# Supplementary material for: Human Hematopoietic Stem Cells Enhance Maturational Differentiation of hiPSC-Derived Cardiomyocytes on Xeno-Free MatriClone-Plastic via EGFR/MAPK/ERK Signaling Pathway
Source: Pharmaceuticals (Basel). 2026 Jun 22;19(6):964. doi: 10.3390/ph19060964 (PMC13304730; doi:10.3390/ph19060964)
Supplement: Supplementary file 1 [file pharmaceuticals-19-00964-s001.zip › Supplementary material1.pdf]

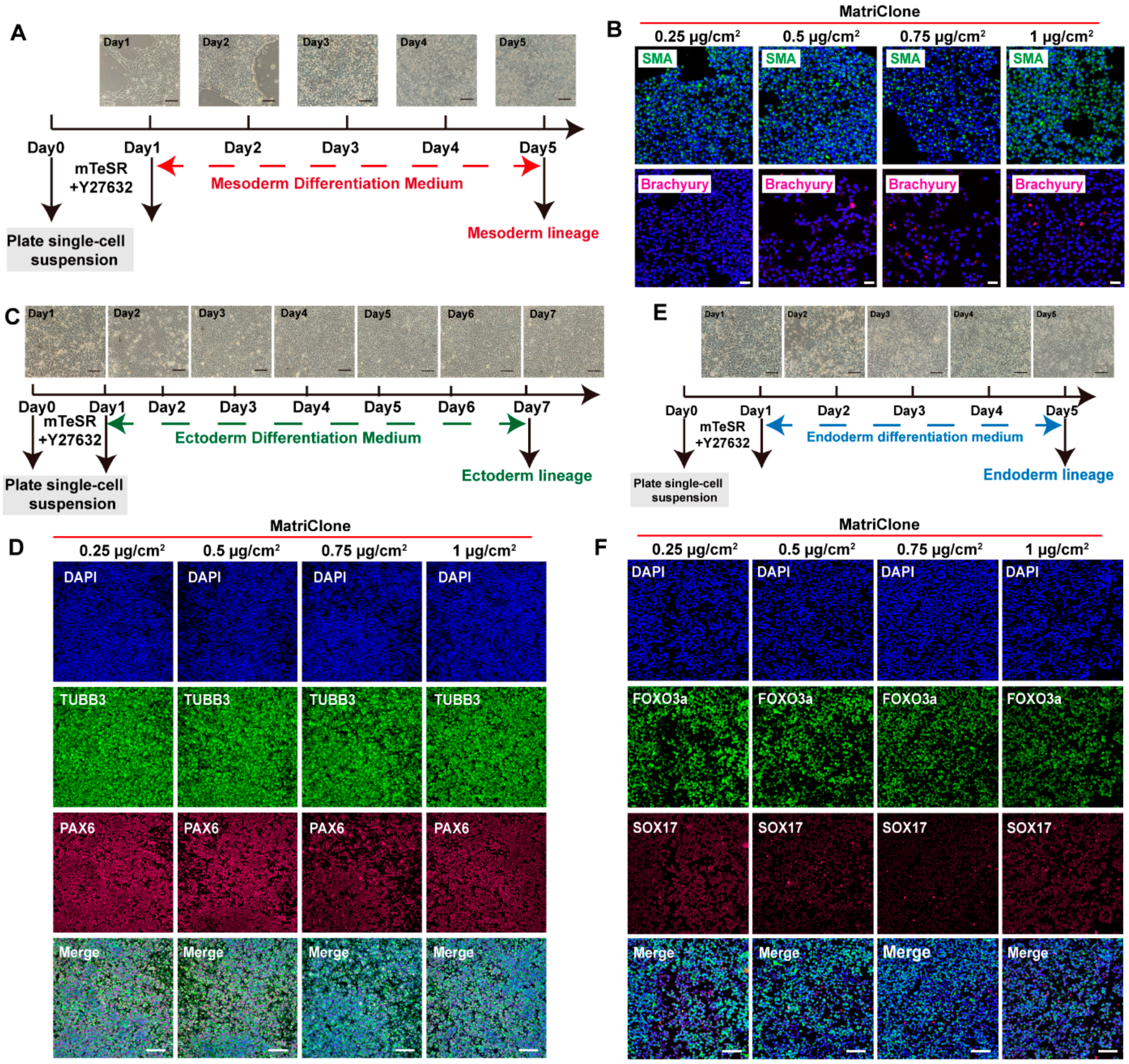

Figure S1. Differentiation and identification of all three germ layers in MatriClone at different densities. (**A**) Schematic representation of the differentiation procedure and sequential morphological changes (Day 0–5) of hiPSC differentiation into mesoderm lineages, Scale bars = 100 μm; (**B**) Representative immunofluorescence images of the mesodermal lineage specific markers SMA and Brachyury in cells on MatriClone at densities ranging from 0.25-1 μg/cm^2, Scale bars = 25 μm. (**C**) Schematic representation of the differentiation procedure and sequential morphological changes (Day 0–7) of hiPSC differentiation into ectoderm lineages, Scale bars = 100 μm; (**D**) Representative immunofluorescence images of the ectoderm lineage specific markers TUBB3 and PAX6 in cells on MatriClone at densities ranging from 0.25-1 μg/cm^2, Scale bars = 50 μm; (**E**) Schematic representation of the differentiation procedure and sequential morphological changes (Day 0–5) of hiPSC differentiation into endoderm lineages, Scale bars = 100 μm; (**F**) Representative immunofluorescence images of the endoderm lineage specific markers FOXO3a and SOX17 in cells on MatriClone at densities ranging from 0.25-1 μg/cm^2, Scale bars = 50 μm.

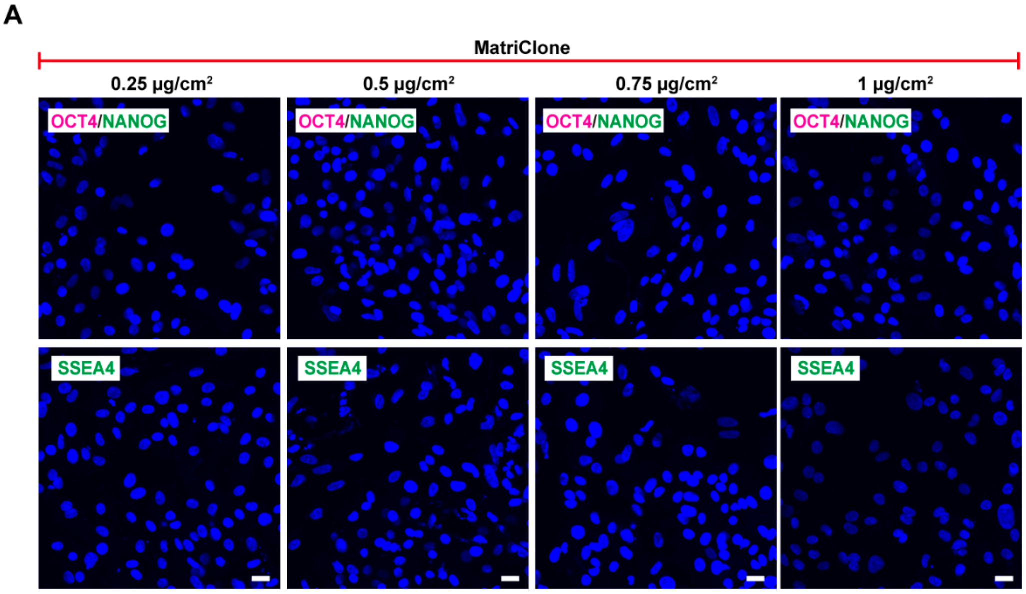

Figure S2. Pluripotency identification of hiPSC-CMs in MatriClone at different densities. (**A**) Representative immunofluorescence images of NANOG, OCT4 and SSEA4 expression in hiPSC-CMs on MatriClone at densities ranging from 0.25-1 μg/cm^2. Scale bars = 25 μm.

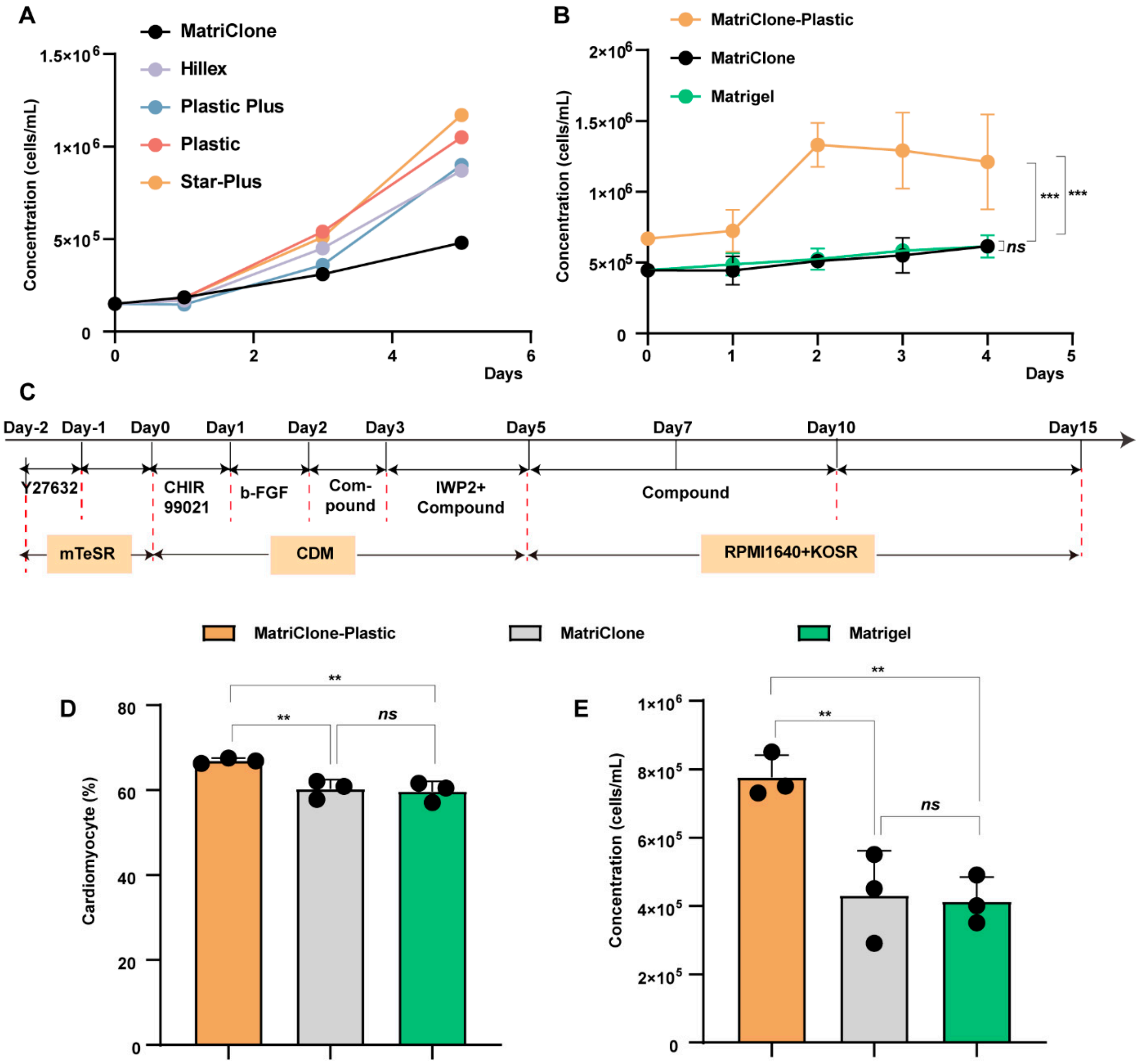

Figure S3. Cardiac differentiation on 1 μg/cm² MatriClone-Plastic. (**A**) The amounts of hiPSCs on four microcarriers coated with 1 μg/cm² MatriClone were detected on the 1st, 3rd and 5th day after cell seeding (n = 1); (**B**) The amounts of hiPSCs on Matrigel, 1 μg/cm² MatriClone and 1 μg/cm² MatriClone-Plastic were detected every day (n = 3); (**C**) Schematic representation of the differentiation procedure of hiPSC differentiation into CMs induced by the saponin+ compound on 1 μg/cm² MatriClone-Plastic; (**D**) Flow cytometry was used to detect the proportion of cTnI-expressing cells on Matrigel, 1 μg/cm² MatriClone and 1 μg/cm² MatriClone-coated Plastic (n = 3); (**E**) The amounts of cells on Matrigel, 1 μg/cm² MatriClone and 1 μg/cm² MatriClone-Plastic were detected on the 15th day of CM differentiation (n = 3). All data were expressed as mean ± SD. Data in (**B**), (**D**), and (**E**) were analyzed by one-way ANOVA. ** $P < 0.01$, *** $P < 0.001$, ns, no significance.

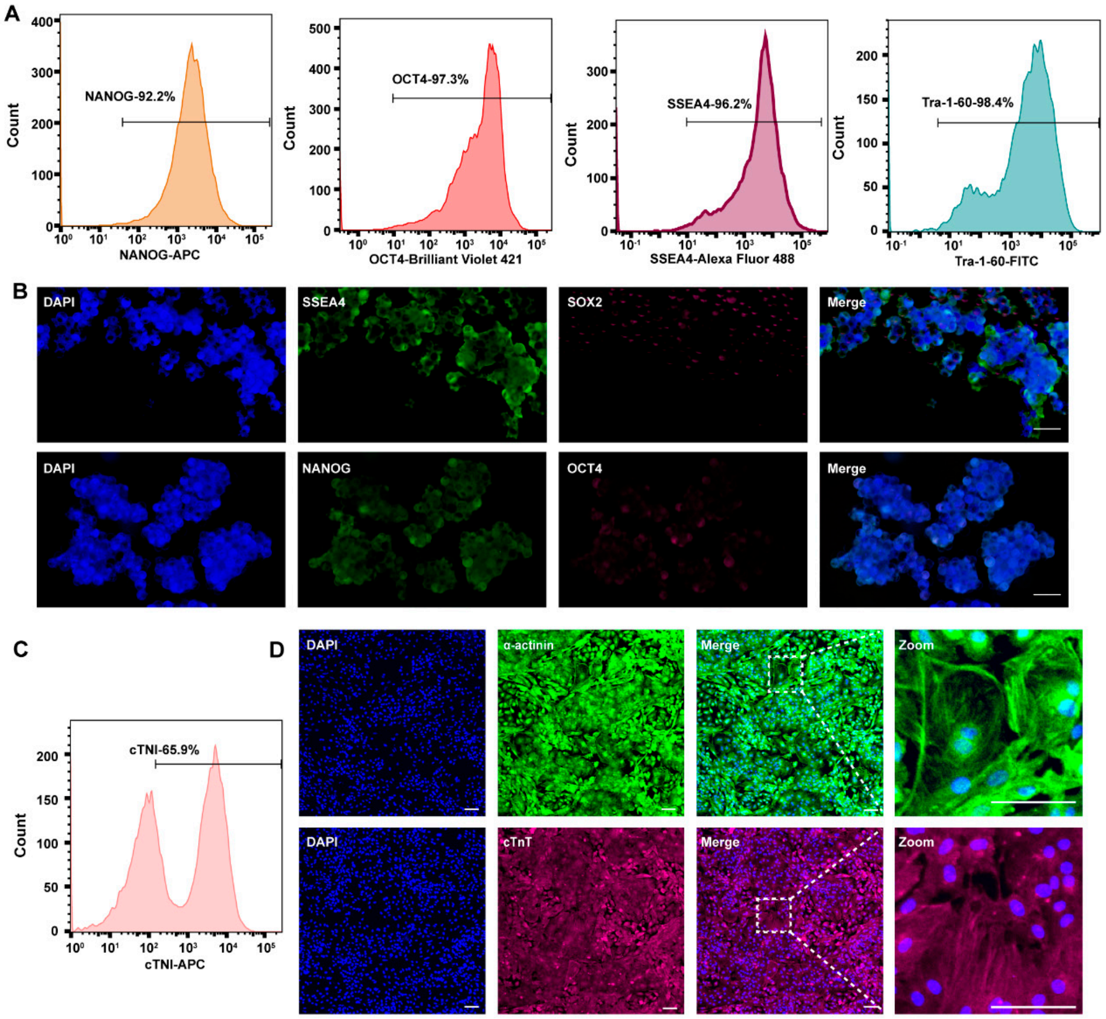

Figure S4. Expansion and cardiac differentiation of hiPSC lines from B1 (derived from blood cells) on 1 μg/cm² MatriClone-Plastic. (A) Representative histograms of hiPSC lines from B1 (derived from blood cells) showed the proportion of NANOG, OCT4, SSEA4 and Tra-1-60-expressing cells for ten consecutive passages on 1 μg/cm² MatriClone-Plastic; **(B)** Representative immunofluorescence images of the pluripotency markers expression in hiPSC lines from B1 (derived from blood cells) on 1 μg/cm² MatriClone-Plastic, Scale bars =1 mm; **(C)** Representative histogram of hiPSC-CMs showed the proportion of cTnI-expressing cells in Day 15 hiPSC-CMs on 1 μg/cm² MatriClone-Plastic; **(D)** Representative immunofluorescence images of CM markers α-actinin and cTNT on 1 μg/cm² MatriClone-Plastic, Scale bars = 200 μm.

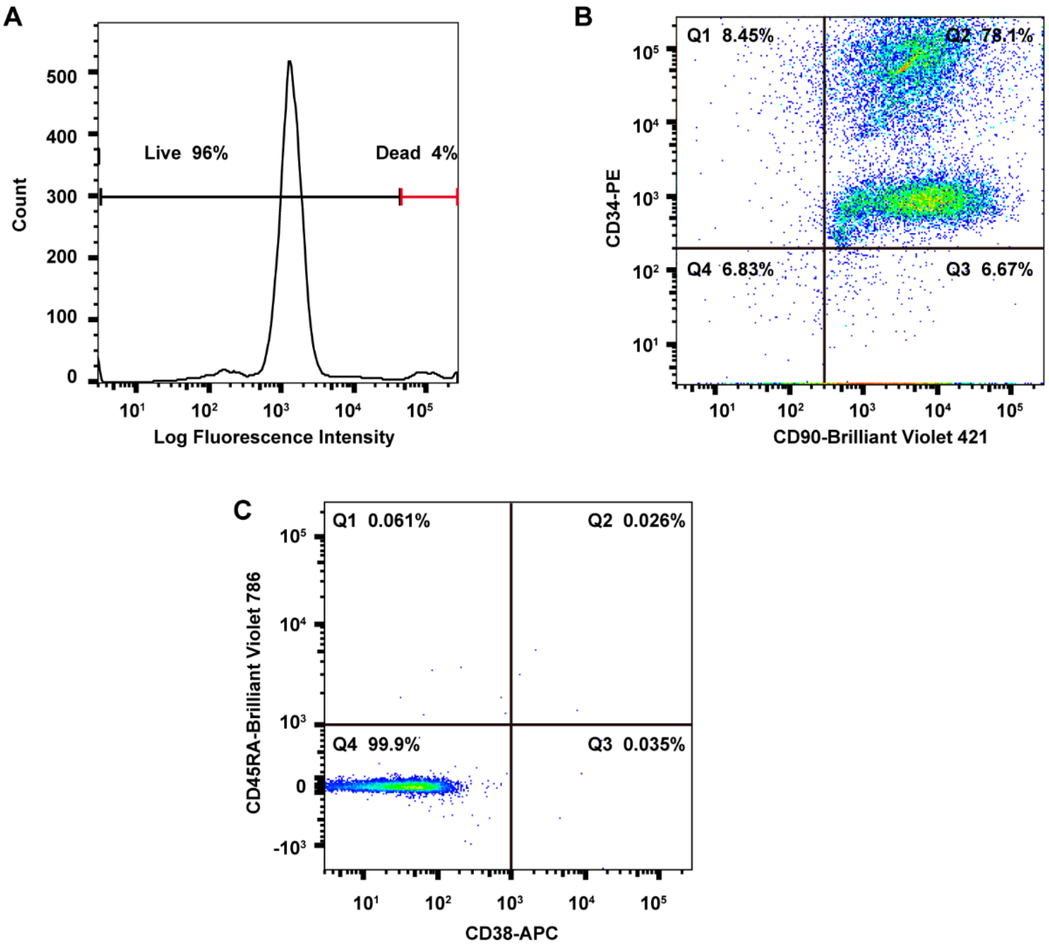

Figure S5. hHSC identification. (A) Representative flow cytometry analysis of Zombie NIR dyed hHSCs; **(B)** Representative flow cytometry analysis of hHSC specificity marker expression, The results showed that the proportions of hHSCs with double-positive expression of CD90 and CD34 was 78.1%; **(C)** Representative flow cytometry analysis of hHSC specificity marker expression, The results showed that the proportion of hHSCs with double-negative expression of CD38 and CD45RA was 99.9%.

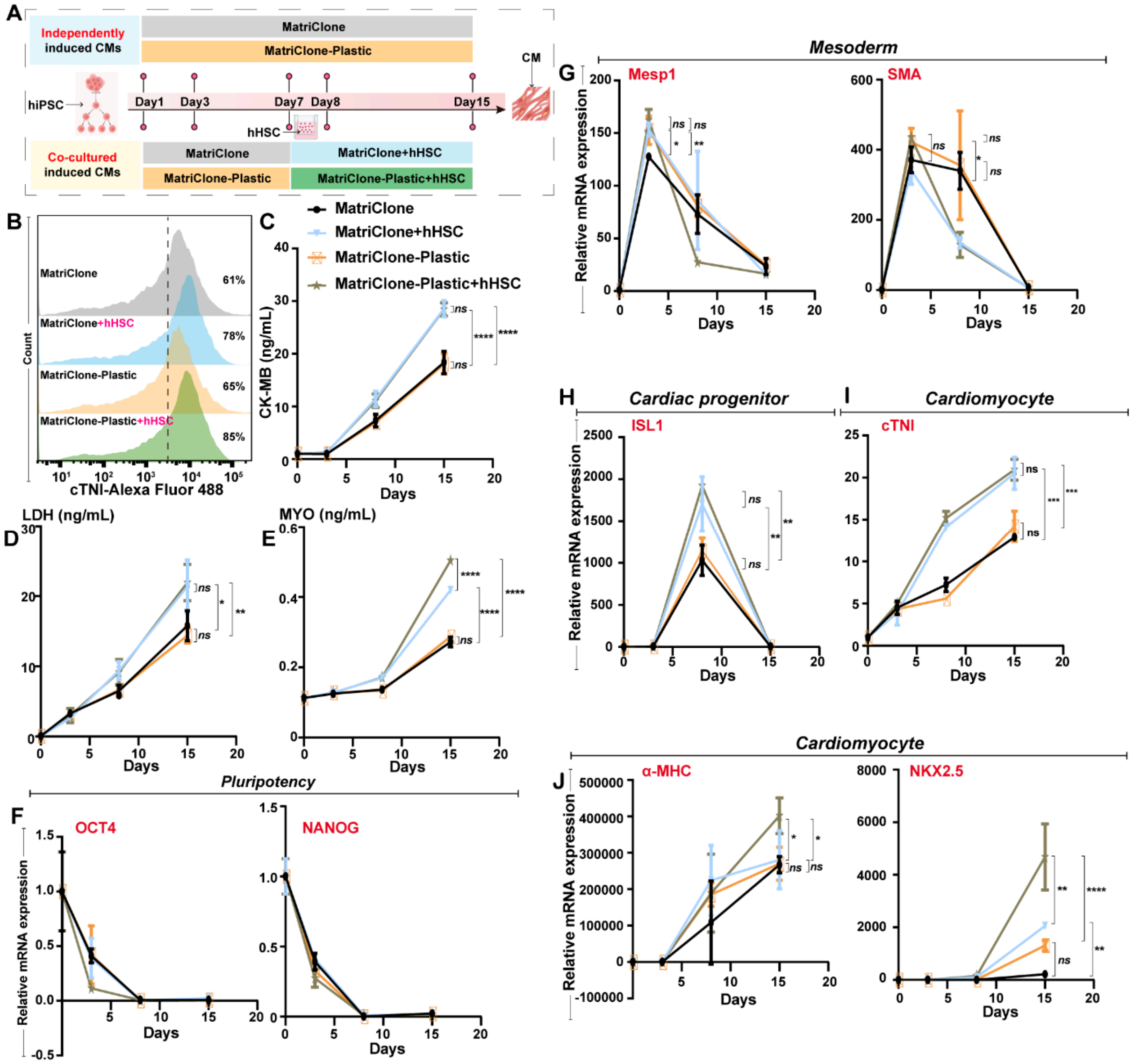

Figure S6. Comparison of characterization and structure of co-cultured and independently induced CMs. (A) Schematic diagram of the experiment for co-cultured and independently induced CMs; **(B)** Representative histogram of hiPSC-CMs showed the proportion of cTnI-expressing cells in Day 15 hiPSC-CMs; **(C-E)** The levels of CK-MB, LDH and MYO of cells in each group were detected by ELISA on the 3rd, 8th and 15th day of cardiac differentiation (n = 4); **(F)** qPCR analysis was used to characterize undifferentiated cell marker genes OCT4 and NANOG in each group on the3rd, 8th and 15th day of cardiac differentiation (n = 3-4) ; **(G)** qPCR analysis was used to characterize mesoderm markers SMA and Mesp1 in each group on the 3rd, 8th and 15th day of cardiac differentiation (n = 3-4); **(H)** qPCR analysis was used to characterize cardiac progenitor markers ISL1 in each group on the 3rd, 8th and 15th day of cardiac differentiation (n = 3-4); **(I-J)** qPCR analysis was used to characterize CM markers cTNI, α-MHC and NKX2.5 in each group on the 3rd, 8th and 15th day of cardiac differentiation (n = 3-4). All data were expressed as mean ± SD. The MatriClone group was used as negative control. Normality testing was performed using Shapiro-Wilk testing before selecting statistical tests as appropriate. Data in **(C)** to **(E)**, **(G)** to **(J)** were analyzed by one-way ANOVA. $^{*}P < 0.05$, $^{**}P < 0.01$, $^{***}P < 0.001$, $^{****}P < 0.0001$, ns, no significance.

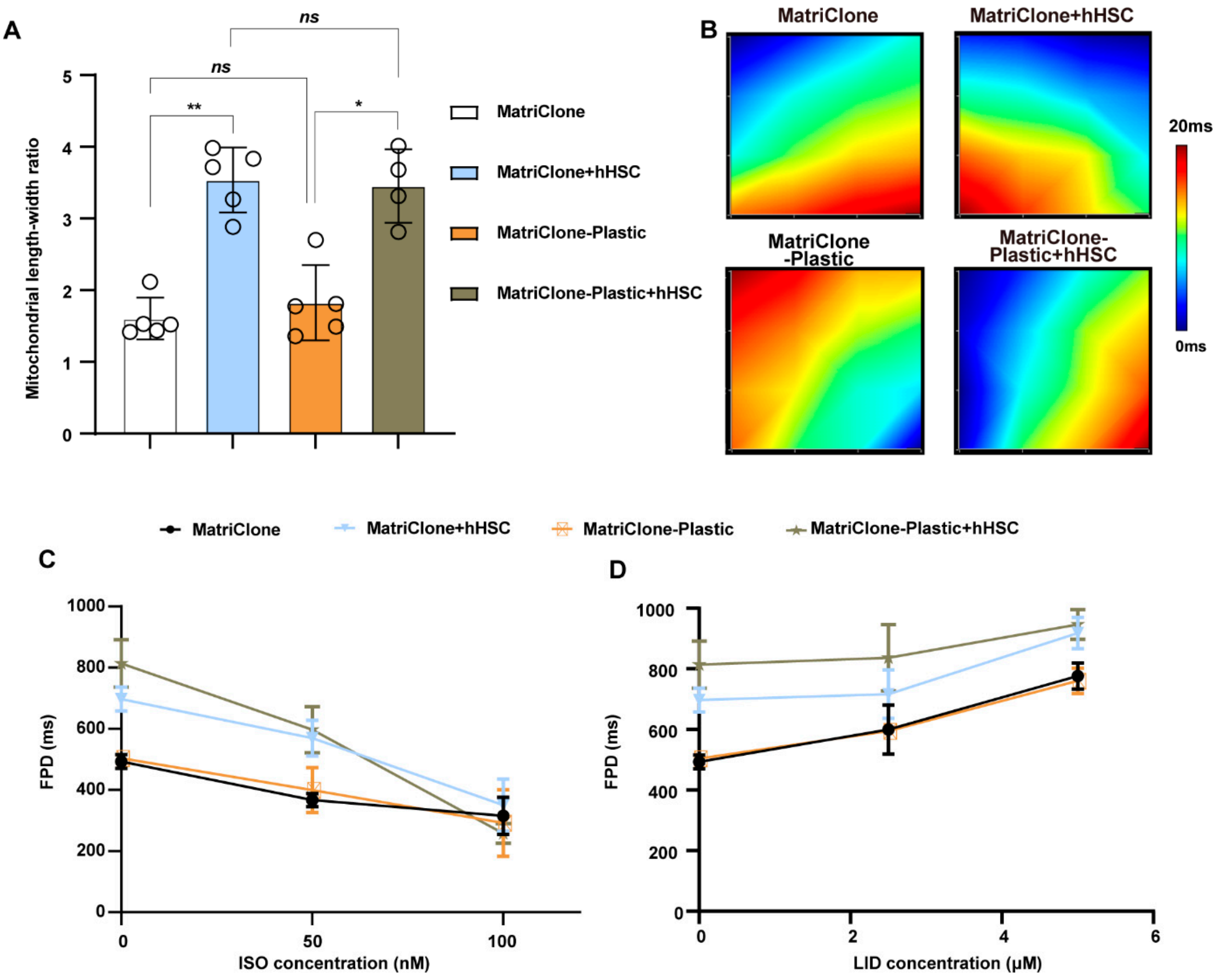

Figure S7. Comparison of structure and function of co-cultured and independently induced CMs. (A) The quantification of mitochondrial length-width ratio in (**Figure. 4A**) by Image J (n = 5); **(B)** Representative conduction heatmaps that provided an intuitive way to visualize the direction of hiPSC-CM beating of the four comparison groups. The blue region represented the origin of the beat (start electrode); **(C)** The quantification of FPD after added ISO in (**Figure. 4I**) by Image J (n = 3); **(D)** The quantification of FPD after added LID in (**Figure. 4I**) by Image J (n = 3). All data were expressed as mean ± SD. Data in **(A)** were analyzed by nonparametric testing. $^{*}P < 0.05$, $^{**}P < 0.01$, ns, no significance.

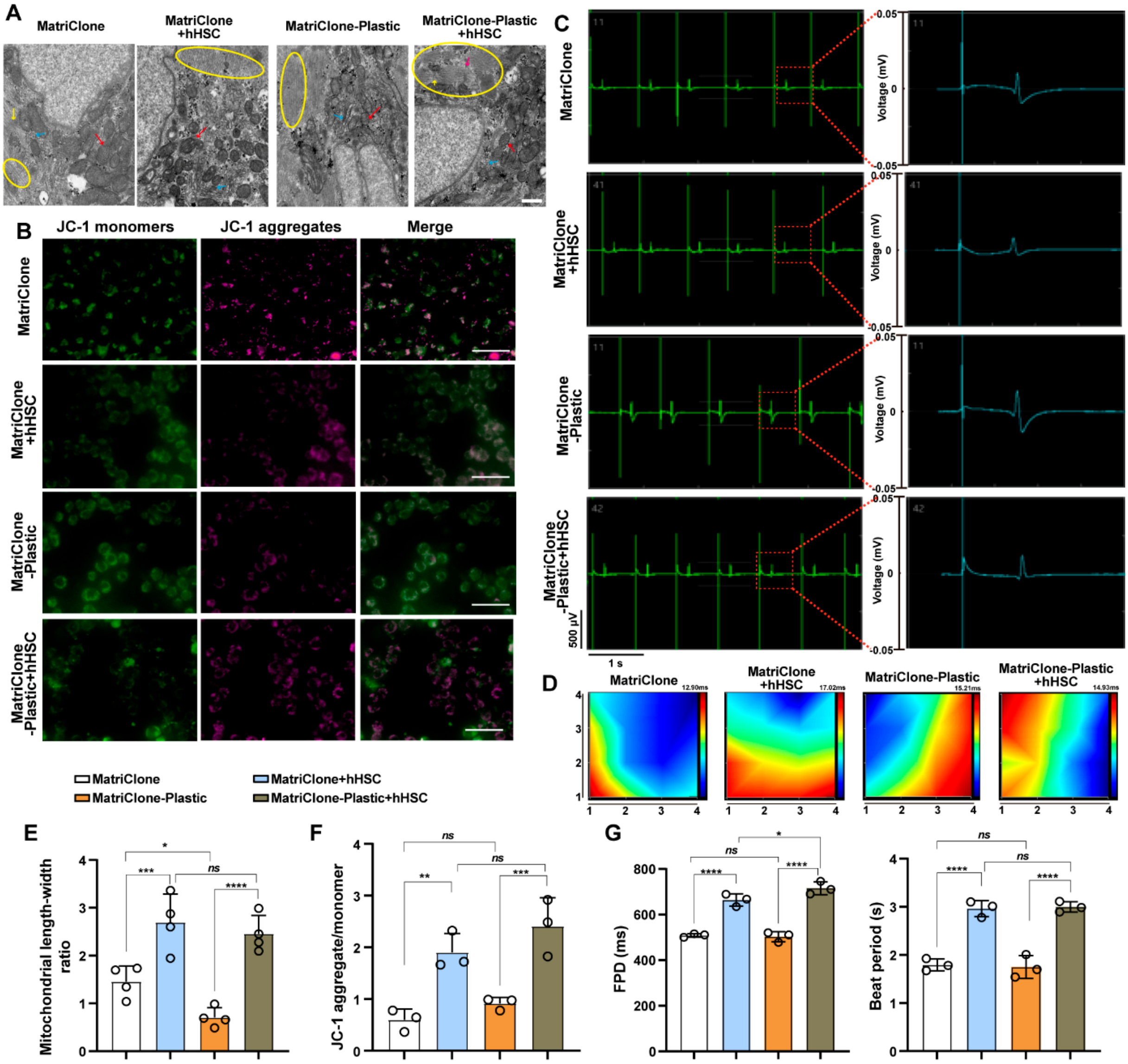

Figure S8. Co-culture of hiPSCs line of B1 (derived from blood cells) and Human Cord Blood Derived HSCs for cardiac differentiation. **(A)** Representative electroscopic images of CMs in each group, Red arrows indicate mitochondria, Blue arrows indicate sarcoplasmic reticulum, Yellow circles indicate myofilaments, Yellow arrow indicated H line, Magenta arrow indicated intercalated disk, Symbols (*) indicate lipid droplets, Scale bars = 2 μm; **(B)** Representative images of JC-1 staining of hiPSC-CMs of the four comparison groups on the 15th day, MMP of living hiPSC-CMs was detected by JC-1 fluorescent probe, Scale bars = 200 μm; **(C)** Representative continuous waveform of one electrode on a single well by MEA, Spontaneous beating hiPSC-CMs were confirmed by the extracellular FP recorded on the 6th day after cell seeding; **(D)** Representative conduction heatmaps that provided an intuitive way to visualize the direction of hiPSC-CM beating of the four comparison groups, The blue region represented the origin of the beat (start electrode); **(E)** The quantification of mitochondrial length-width ratio in (**A**) by Image J (n = 3); **(F)** The fluorescence intensity of JC-1 staining in (**B**) were quantified by Image J (n = 3); **(G)** The differences in beat period and FPD in cultured hiPSC-CMs of the four comparison groups were detected by MEA (n = 3). All data were expressed as mean ± SD. Normality testing was performed using Shapiro-Wilk testing before selecting statistical tests as appropriate. Datas were analyzed by one-way ANOVA. $^{*}P < 0.05$, $^{**}P < 0.01$, $^{***}P < 0.001$, $^{****}P < 0.0001$, ns, no significance.

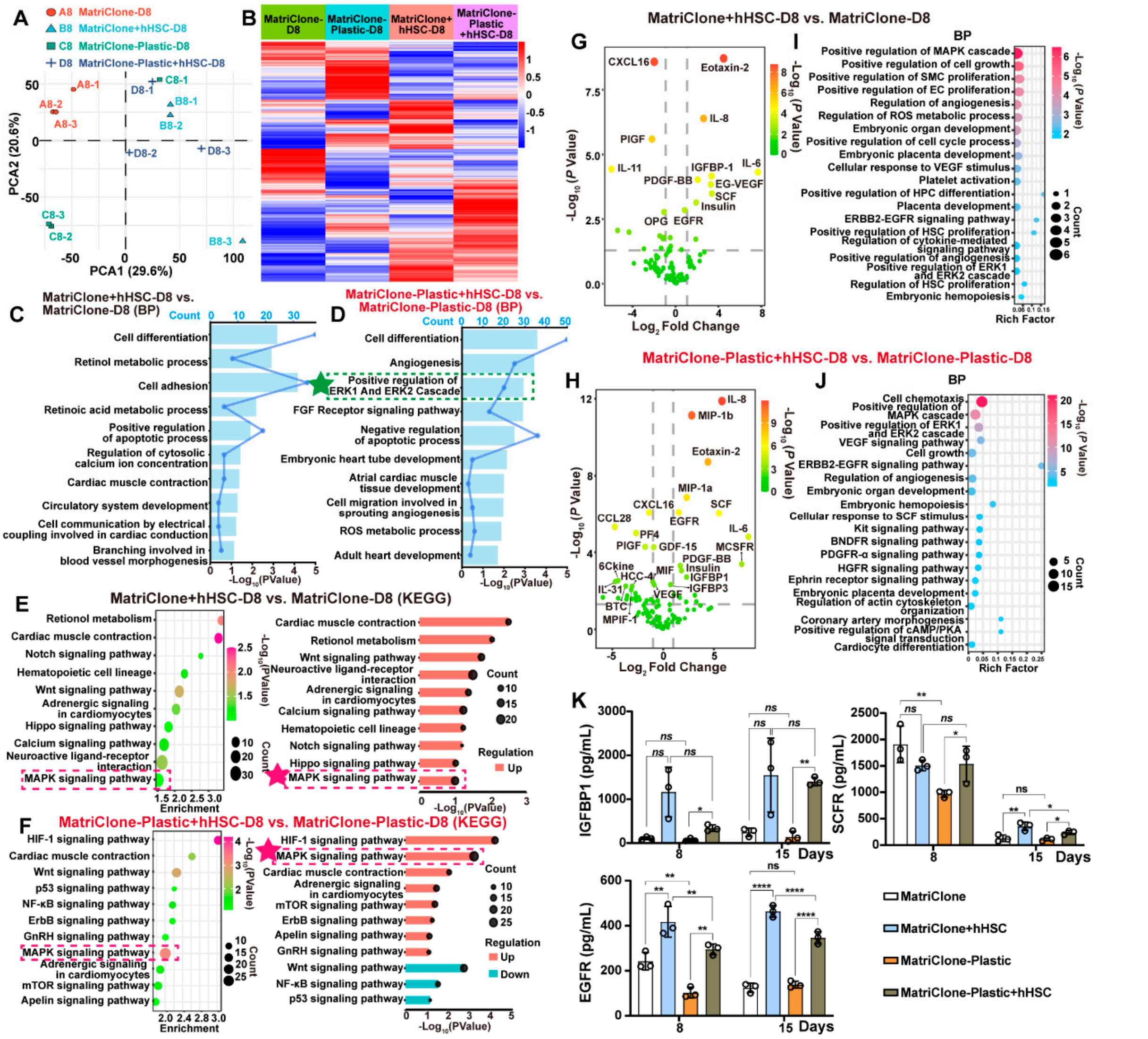

Figure S9. Comparison of gene expression profiles by RNA-seq and cytokine-related proteins in supernatants by cytokine array of co-cultured and independently induced CMs on the 8th day. (A) PCA was performed based on the common genes of MatriClone-D8, MatriClone+hHSC-D8, MatriClone-Plastic-D8 and MatriClone-Plastic+hHSC-D8; **(B)** The clustered heatmap showed the gene expression of MatriClone-D8, MatriClone+hHSC-D8, MatriClone-Plastic-D8 and MatriClone-Plastic+hHSC-D8; **(C)** BPs in GO enrichment analysis of DEGs between MatriClone+hHSC-D8 and MatriClone-D8; **(D)** BPs in GO enrichment analysis of DEGs between MatriClone-Plastic+hHSC-D8 and MatriClone-Plastic-D8; **(E)** KEGG enrichment analysis of DEGs between MatriClone+hHSC-D8 and MatriClone-D8; **(F)** KEGG enrichment analysis of DEGs between MatriClone-Plastic+hHSC-D8 and MatriClone-Plastic-D8; **(G)** Volcano plots for of the cytokine array in supernatants of MatriClone+hHSC-D8 and MatriClone-D8; **(H)** Volcano plots for of the cytokine array in supernatants of MatriClone-Plastic+hHSC-D8 and MatriClone-Plastic-D8; **(I)** BPs in GO enrichment analysis of DEPs between MatriClone+hHSC-D8 and MatriClone-D8; **(J)** BPs in GO enrichment analysis of DEPs between MatriClone-Plastic+hHSC-D8 and MatriClone-Plastic-D8; **(K)** The expressions of IGFBP1, SCFR, and EGFR in the supernatants of the four comparison groups on the 8th and 15th day of cardiac differentiation (n = 3). All data were expressed as mean ± SD. Normality testing was performed using Shapiro-Wilk testing before selecting statistical tests as appropriate. Data in **(K)** were analyzed by one-way ANOVA. * $P < 0.05$, ** $P < 0.01$, **** $P < 0.0001$, ns, no significance.

Table S1. The chemical characterization of four microcarriers

Name	Cat no.	Material	Relative Density	Size (μm)	Surface Area (cm^2/g)
Hillex	H-170-020	Modified polystyrene, cationic-charge	1.080-1.150	150-210	515cm^2
Plastic Plus	PP-221-020	Cross-linked polystyrene, cationic-charge	1.022-1.030	125-212	360cm^2
Plastic	P-221-020	Cross-linked polystyrene	1.022-1.030	125-212	360cm^2
Star-Plus	SP-221-020	Cross-linked modified polystyrene, cationic-charge	1.020-1.030	125-212	360cm^2

Summary of a preliminary screening of 4 different available microcarriers.

Table S2. Antibodies used in immunofluorescence analyses.

Target	Host	Supplier	Cat no.	Dilution
OCT4	Rabbit	Cell Signaling	#2750	1:200
NANOG	Mouse	Cell Signaling	#4893	1:1000
SSEA4	Mouse	Abcam	ab16287	1:100
SOX2	Rabbit	Proteintech	11064-1-AP	1:200
cTNT	Rabbit	Bioss	bs-10614R	1:30
α-actinin	Mouse	Proteintech	66895-1-Ig	1:500
PAX6	Rabbit	Proteintech	12323-1-AP	1:50
TUBB3	Mouse	Proteintech	66375-1-Ig	1:200
Brachyury	Rabbit	Abcam	ab209665	1:1000
SMA	Mouse	Santa Cruz	sc-53142	1:200
FOXO3a	Mouse	Cell Signaling	#99199	1:800
SOX17	Rabbit	Proteintech	24903-1-AP	1:200
EGFR	Rabbit	Abcam	ab52894	1:100
Goat Anti-Mouse IgG H&L (Alexa Fluor® 488)	Goat	Abcam	ab150117	1:500
Goat Anti-Rabbit IgG H&L (Alexa Fluor® 647)	Goat	Abcam	ab150083	1:500

Table S3. Antibodies used in flow cytometry.

Target	Host	Supplier	Cat no.
OCT4	Mouse	BioLegend	653712
NANOG	Rabbit	Proteintech	CL647-14295
SSEA4	Mouse	BioLegend	330411
TRA-1-60	Mouse	BD Pharmingen	560876
cTNI	Mouse	BD Pharmingen	564409
cTNI	Rabbit	Abcam	ab52862
Goat Anti-Rabbit IgG H&L (Alexa Fluor® 488)	Goat	Abcam	ab150077
Zombie NIR™		BioLegend	423105
CD34	Mouse	BioLegend	378603
CD90	Mouse	BioLegend	328121
CD45RA	Mouse	BD Pharmingen	563870
CD38	Mouse	BD Pharmingen	560980

Table S4. Quantitative PCR primer list.

Name	Forward primer sequence	Reverse primer sequence
Human-Mesp1	CTGGCTCTGTTGGAGACCTG	ACCACTTCGAAGGTGCTGAG
Human-SMA	AAAAGACAGCTACGTGGGTGA	GCCATGTTCTATCGGGTACTTC
Human-ISL1	GCGGAGTGTAATCAGTATTTGGA	GCATTTGATCCCGTACAACCT
Human-NKX2.5	GAGCCGAAAAGAAAGCCTGAA	CACCGACACGTCTCACTCAG
Human-GATA4	CGACACCCCAATCTCGATATG	GTTGCACAGATAGTGACCCGT
Human-α-MHC	GCCCTTTGACATTCGCACTG	GGTTTCAGCAATGACCTTGCC
Human-cTNI	TTTGACCTTCGAGGCAAGTTT	CCCGGTTTTCCTTCTCGGTG
Human-PAX6	TGGGCAGGTATTACGAGACTG	ACTCCCGCTTATACTGGGCTA
Human-TUBB3	GGCCAAGGGTCACTACACG	GCAGTCGCAGTTTTCACACTC
Human-Brachyury	ACGACAACGGCCACATTATTC	CCTCGGCATATTTCTCGCTATCT
Human-SMA	AAAAGACAGCTACGTGGGTGA	GCCATGTTCTATCGGGTACTTC
Human-FOXO3a	CGGACAAACGGCTCACTCT	GGACCCGCATGAATCGACTAT
Human-SOX17	GTGGACCGCACGGAATTTG	GGAGATTCACACCGGAGTCA
Human-NANOG	TTTGTGGGCCTGAAGAAAACT	AGGGCTGTCCTGAATAAGCAG
Human-OCT4	CTGGGTTGATCCTCGGACCT	CCATCGGAGTTGCTCTCCA
Human-Akt	AGCGACGTGGCTATTGTGAAG	GCCATCATTCTTGAGGAGGAAGT
Human-MEK	CAATGGCGGTGTGGTGTTC	GATTGCGGGTTTGATCTCCAG
Human-ERK1	CTACACGCAGTTGCAGTACAT	CAGCAGGATCTGGATCTCCC
Human-ERK2	TACACCAACCTCTCGTACATCG	CATGTCTGAAGCGCAGTAAGATT
Human-RSK1	CAGTGGGCACCTGTATGCTAT	ACGAATGGGTGATTTACATCAGC
Human-RSK2	ACTTGGAAGATTTTCGTGGGC	CGGAACCATACCCTTGGTGG

Human-actin	CATGTACGTTGCTATCCAGGC	CTCCTTAATGTCACGCACGAT

Table S5. Cell lines.

Name	Supplier	Passage no.
hiPSC-U1	Beijing Saibei Biotechnology Co., Ltd	20-30 passages
hiPSC-B1	Beijing Saibei Biotechnology Co., Ltd	20-30 passages
Human Bone Marrow Derived HSCs	Lonza	Primary Generation
Human Cord Blood Derived HSCs	Xiamen Immocell Biotechnology Co. Ltd	Primary Generation
